# Cross-species multiple environmental stress responses: An integrated approach to identify candidate genes for multiple stress tolerance in sorghum (*Sorghum bicolor* (L.) Moench) and related model species

**DOI:** 10.1371/journal.pone.0192678

**Published:** 2018-03-28

**Authors:** Adugna Abdi Woldesemayat, David M. Modise, Junaid Gemeildien, Bongani K. Ndimba, Alan Christoffels

**Affiliations:** 1 South African Medical Research Council Bioinformatics Unit, South African National Bioinformatics Institute, University of the Western Cape, Belleville, South Africa; 2 Department of Life and Consumer Sciences, College of Agriculture and Environmental Sciences, University of South Africa, Science Campus, Florida, Johannesburg, South Africa; 3 Department of Agriculture and Animal Health, College of Agriculture and Environmental Sciences, University of South Africa, Science Campus, Florida, Johannesburg, South Africa; 4 Department of Biotechnology, University of the Western Cape, Cape Town, Western Cape, South Africa; 5 Agricultural Research Council, Infruitech-Nietvoorbij, Stellenbosch, South Africa; University of Toronto, CANADA

## Abstract

**Background:**

Crop response to the changing climate and unpredictable effects of global warming with adverse conditions such as drought stress has brought concerns about food security to the fore; crop yield loss is a major cause of concern in this regard. Identification of genes with multiple responses across environmental stresses is the genetic foundation that leads to crop adaptation to environmental perturbations.

**Methods:**

In this paper, we introduce an integrated approach to assess candidate genes for multiple stress responses across-species. The approach combines ontology based semantic data integration with expression profiling, comparative genomics, phylogenomics, functional gene enrichment and gene enrichment network analysis to identify genes associated with plant stress phenotypes. Five different ontologies, viz., Gene Ontology (GO), Trait Ontology (TO), Plant Ontology (PO), Growth Ontology (GRO) and Environment Ontology (EO) were used to semantically integrate drought related information.

**Results:**

Target genes linked to Quantitative Trait Loci (QTLs) controlling yield and stress tolerance in sorghum (*Sorghum bicolor* (L.) Moench) and closely related species were identified. Based on the enriched GO terms of the biological processes, 1116 sorghum genes with potential responses to 5 different stresses, such as drought (18%), salt (32%), cold (20%), heat (8%) and oxidative stress (25%) were identified to be over-expressed. Out of 169 sorghum drought responsive QTLs associated genes that were identified based on expression datasets, 56% were shown to have multiple stress responses. On the other hand, out of 168 additional genes that have been evaluated for orthologous pairs, 90% were conserved across species for drought tolerance. Over 50% of identified maize and rice genes were responsive to drought and salt stresses and were co-located within multifunctional QTLs. Among the total identified multi-stress responsive genes, 272 targets were shown to be co-localized within QTLs associated with different traits that are responsive to multiple stresses. Ontology mapping was used to validate the identified genes, while reconstruction of the phylogenetic tree was instrumental to infer the evolutionary relationship of the sorghum orthologs. The results also show specific genes responsible for various interrelated components of drought response mechanism such as drought tolerance, drought avoidance and drought escape.

**Conclusions:**

We submit that this approach is novel and to our knowledge, has not been used previously in any other research; it enables us to perform cross-species queries for genes that are likely to be associated with multiple stress tolerance, as a means to identify novel targets for engineering stress resistance in sorghum and possibly, in other crop species.

## Background

Identification of genetic determinants for multi-stress responses is regarded as the most reliable approach towards improving crop production and yield stability. However, dissection of genetic determinants for normal biological function under plant stresses such as drought is the most daunting task in plant genetics due to complexity in stress associated perturbations that may elicit complex networks and cross talk, and the multifactorial patterns of quantitative trait inheritance. For such features, identification of genes for drought and related stress tolerance, particularly for use in breeding, remains a common challenge in most cereal genomes [1]. Of note, an accelerated world population growth coupled with a concurrently aggravated global warming and climate change and the associated unpredictable effects of recurrent drought have become a critically important issue for global food security. To mitigate this alarming concern and meet the changing global food requirements, crop adaptation to the changing environment and productivity under multi-environmental stresses must be improved over the coming years.

Previous studies on sorghum and related cereals were focused on gene-phenotype modeling at crop level mainly with regards to the challenges of crop management and genotype-environment interactions with respect to modern breeding approaches [2]. Recent works have investigated the impact and interaction of simultaneous biotic and abiotic stresses on plant performance [3,4], including shared and unique responses as a physio-molecular mechanism [5]. Other studies have been conducted in attempt to improve productivity under environmental stresses such as drought [6–8], however, appropriate strategies have not yet been adopted to successfully address the issues of the complex crop traits related to multiple tolerance. To the best of our knowledge, none of these studies were targeted for multiple stress response across species to enhance crop productivity. Identification of plant genes linked to traits responding to stress combination across species is of paramount importance for crop improvement and yield stability. In the present approach, functional ontology was used as the basis for query building and semantic integration of data, allowing identification of genes regulating complex traits using orthology based comparative genomics and phylogenomics, gene expression profiling, biological networks and data mining from known biological information.

Orthology related comparative genomic analysis has been a useful tool in identification of functionally equivalent orthologs across species. It provides transitive association of experimental and biological information inferring the extent of evolutionary conservation of this information between species [9]. However, caution should be exercised when comparing sequence similarity alone for estimating functional conservation in plants, due to frequent gene duplication in plant genomes [9,10]. Nevertheless, a recent study has shown that similarities in the pattern of expression profiles between orthologs are likely to be instrumental in predicting conserved functional orthologs even after gene duplication [10]. In addition, molecular evolutionary studies have enabled the investigation of new emerging complex traits of functional ortholog groups by combining comparative genomics and phylogenomics with co-expression of gene networks in plants [11]. Current advances in the gene expression profiling have contributed to the identification of key plant genes involved in the wider range of stress responses [12,13]. The advantage of using gene expression profiling in mining genes for plant stress response relies on the causal linkage established between gene expression and stress tolerance, because the former represents a quantifiable intermediate phenotype that can reveal association between molecular perturbations and stress phenotypes [14].

Probably the most difficult task in gene identification for stress tolerance is to scale down a large-set of genes into a potentially promising list of target genes. An integrated functional ontology approach, as the basis for gene association method, using gene set enrichment tools for finding complex traits is deemed to be the most promising approach to obtain biologically relevant and concise number of genes involved in drought and related stress responses [15,16]. This approach employs multiple options for identification of key physiological and developmental traits that relate to gene-phenotype association under stresses.

The plant ontology-based identification of complex traits using association analysis includes a wide spectrum of interrelated components. Among the 5 different plant ontologies that are widely used to semantically integrate data, the Gene Ontology (GO) is the first that our approach uses to identify candidate genes for drought related tolerance. Gene Ontology is a well-defined and structured shared knowledge in 3 interrelated but non-overlapping domains of molecular biology, namely biological process (BP), molecular function (MF) and cellular component (CC) which are all attributes of genes, gene products or gene-product groups [17,18]. These domains represent a biological aspect to which the gene or gene product contributes, a biochemical activity of a gene product and the place in the cell where a gene product is active, respectively. The GO also deals with gene-centered information such as gene-gene relationship, association and interaction as well as protein-protein interaction [17,19] and mapping of genes to known GO-terms based on biological functions from all GO-categories.

Trait Ontology (TO), being another component of plant ontologies, represents a structured vocabulary of terms that denote phenotypic traits in plants [20] notably plant height, chlorophyll content and stay green characters. Trait Ontology investigates genes associated with peculiar traits which are characteristically classified into genetic, agronomic, biochemical, physiological and developmental traits. These traits represent categories which are familiar in nature but not distinct and are often complementary [20]. Trait Ontology is employed to resolve such non-distinctiveness, because it allows a ‘one to many’ relationships, thus dealing with gene trait association [21]. Plant Ontology (PO) is the first generic ontological representation of anatomical and morphological structure in all plants [22]. Like TO, it addresses the same problem arising due to inconsistencies in terminologies used to describe plant structure and allows the description of gene association to plant morphological and anatomical structures [23]. Growth Ontology (GRO), on the contrary, provides description of distinct growth and developmental stage contained within plant biology dealing with the gene association with such distinct plant physical growth and developmental differences in tissues groups [24]. Last but not least, Environment Ontology (EO) represents a description of a well-defined growth regimen of a plant [20], and models the association and interaction of genes to different environmental regimes and factors.

All the above ontologies provide distinct descriptions of attributes in association with plant stresses. However, unless the analysis is based on a well designed approach that includes different attributes as comprehensively as possible from all plant ontologies, representative phenotypic information may not be obtained in the identification of genes for stress tolerance. An approach of utilizing all domains and attributed categories that encompass entire plant ontologies was imperative. This in combination with other strategies provides a more representative stress-related information to define and identify multiple stress responsive genes, particularly genetic determinants of the complex drought tolerance.

**Fig 1:**
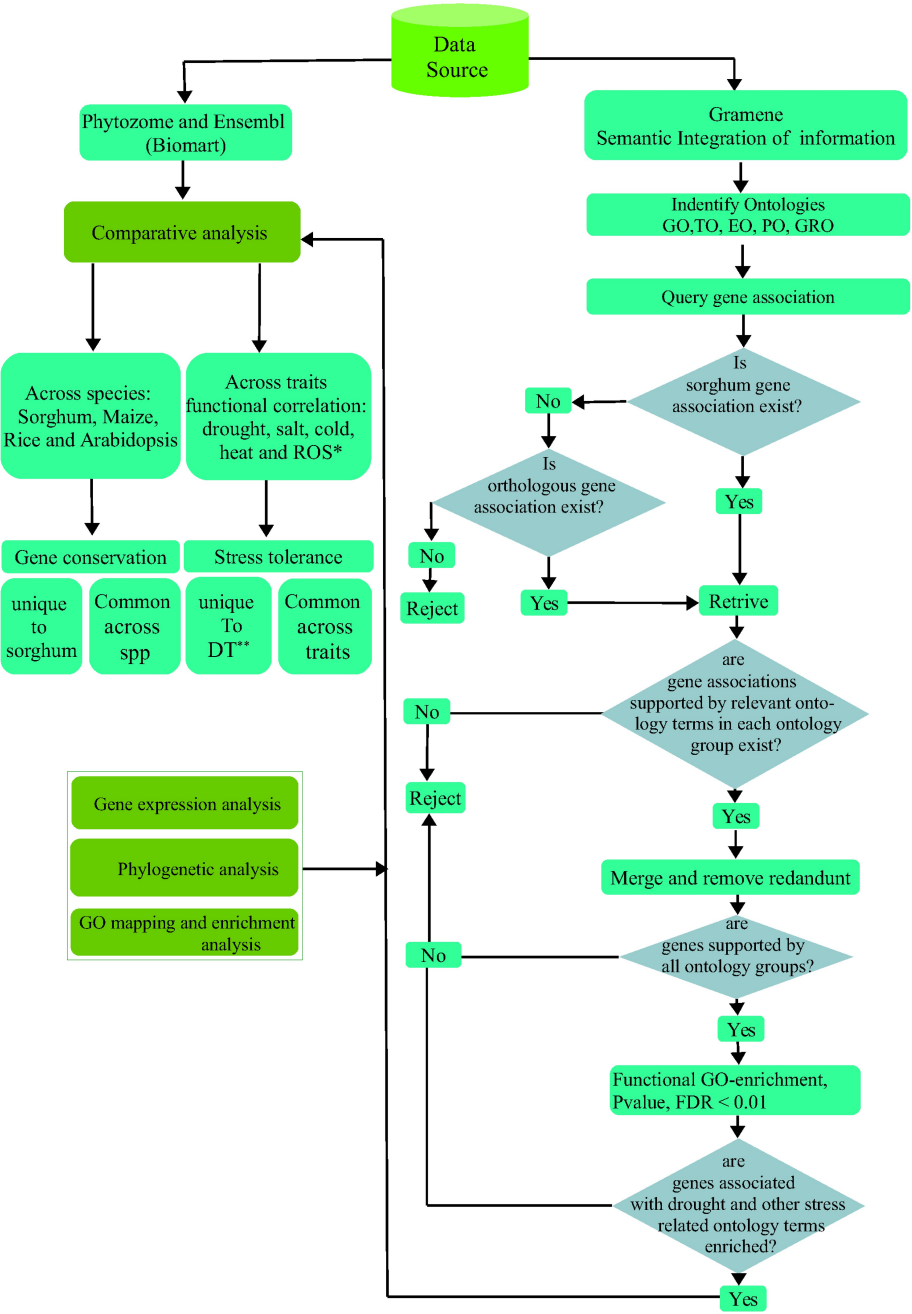
Work-flow for gene-phenotype association across-species and stresses. This figure demonstrates the work flow for the gene-phenotype association in sorghum stress tolerance across species by comparing sorghum drought responsive genes with orthologs in maize, rice and Arabidopsis and across stresses by comparing these genes against salt, cold, heat and oxidative stresses. The Gramene database was used in identification of sorghum genes associated with stress phenotypes based on known stress related ontology terms for each identified plant ontology. Ensembl BioMart was used to get sorghum orthologs having transitive association with known drought regulated functions from related species. The work flow provides a protocol for a step-by-step screening procedure to identify promising gene-sets for multiple stress tolerance across species: 1) The protocol identifies plant ontologies to query genes and detects if the genes belong to the sorghum gene association or to the orthologous group. Where there is no direct sorghum gene association, the protocol looks for orthologous group. Only those genes with these features were retained, and others were discarded. 2) The genes that were not supported by the relevant ontology terms in each ontology group were again rejected and only those with drought and associated ontology terms were screened for the next step. Once merged from all ontology groups, only unique genes were captured by removing the duplicates. 3) Among these, only those which were supported by all ontology groups were used for functional GO enrichment analysis and all others were discarded. 4) Functional GO enrichment analysis based on the P-value, FDR < 0.01 were used to screen the genes associated with stresses under investigation. Only those which satisfied this threshold value were selected as the candidates for the next step. 5) Comparative analysis across species and across traits was undertaken based on the above selected candidates. Sorghum specific and orthologous genes with multi-stress responses were combined with enrichment network and expression profiling for integrative analysis. Sorghum orthologs in other species were selected for which phylogenetic analysis was done. **Key to legend**: * Response to oxidative stress; ** Drought tolerance.

In this study, we therefore introduce a strategy to identify cross-species multiple stress tolerance in plants by combining approaches, for which a technical description is provided in Fig 1. Six distinct approaches including ontology based semantic data integration, functional gene enrichment, expression profiling, gene enrichment network, orthology based comparative genomics and phylogenomics were employed to examine a wide range of complex traits. Furthermore, the approaches allow identification of candidate target genes co-localized within Quantitative Trait Loci (QTLs) involved in the response to multiple stresses and in cross-talk in key signaling pathways in sorghum and other model species. These approaches are universally accepted and employed in gene classification and have empirical evidence of performance in mining stress tolerance determinants [25,26]. This integrated strategy was also employed to identify and classify data and then evaluate using statistical models. As implemented by several statistical metrics [27,28], the functional gene enrichment analysis proved to be useful in screening out the large set of stress related genes into target set of significant responsiveness to multiple stresses. Finally, we investigated the 3 interrelated component parts of drought resistance (DR), namely Drought Tolerance (DT), Drought Avoidance (DA) and Drought Escape (DE). This data represent important experimental information and can be used as the benchmark to study drought and related stress tolerance in other model and non-model crops and in comparative genomic analyses.

## Methods

### Data source and semantic data mining and integration for identification of stress associated genes

Five plant related ontologies, namely GO, TO, PO, GRO, EO were identified using the Gramene [29] and Gene Ontology [30] databases and were used to retrieve and identify sorghum genes that are functionally linked to plant phenotypes and directly or indirectly associated with drought tolerance. The data was uploaded to a local MYSQL database. Semantic queries pertaining to data expressed on the basis of a common vocabulary that leverage semantic information stored in ontologies were used to filter and retrieve the data from relational tables. To determine direct association, drought related ontology terms were first identified for each specified ontology including the number of genes that they represent for sorghum (Fig 1). Where direct association of sorghum gene-trait was not available from the respective ontologies, potential drought tolerant sorghum genes were captured using Ensembl BioMart [31] by transitive association, based on the putative functions of the sorghum gene orthologs in other three related species, namely, maize, rice and Arabidopsis. Ontology mapping was used to represent direct or transitive association of sorghum genes to multiple drought related ontology terms based on orthology functional relationships in maize and rice.

Once sorghum drought associated genes for all ontologies were identified and retrieved, those that were supported by all ontology terms in each ontology group were retained and merged to capture only unique entries. Further, genes supported by all ontology groups were used as an input for functional GO-enrichment (p-value < 0.01) using agriGO [32]. Investigation of gene-phenotype association was based on the correlation of genes to the enriched GO-terms.

### Multiple responses of genes across stresses: Cross-talk and specificity

Using the same initial input as described above, functional correlation of drought responsive genes were compared with genes responsive to other stresses that include salt, cold, heat and oxidative stress. Sorghum drought specific and multiple stress responses were identified using the same procedure described in Fig 1. Genes were selected based on the extent of their association to each environmental stress under particular ontology terms and then filtered based on their enrichment significance level (P-value < 0.05). Where data was lacking for sorghum, closely related orthologs were used to retrieve gene association. Sorghum-rice orthologs were most employed because Gramene data source is comprehensive for rice gene association [20].

### Cross-species comparative analysis: Correlating gene-trait association across species

Comparative analysis were determined based on GO associated drought responsive genes for all GO-domains across species. Ensembl BioMart [31] was used to trace sorghum orthologs in maize, rice and Arabidopsis based on the non-redundant genes identified for GO, TO, PO, GRO and EO with direct or transitive association to sorghum drought tolerance. Sorghum specific genes and those sharing attributes with other species were identified by determining cross-species gene functional association. Sorghum orthologs were compared against each other for specificity and for shared groups of orthology in relation to drought and other stress tolerances across species. Functionally conserved gene groups which are associated with drought tolerance in sorghum were detected by investigating attributes of orthologs in the respective species. Venny [33], an interactive tool for comparing list of genes with Venn Diagrams was used to display and visualize unique and common gene groups.

### Integration of gene trait association with gene differential expression

Sorghum expression data related to drought stress were obtained from the National Center for Biotechnology Information (NCBI), Expression Omnibus (GEO) database [34], accession number GSE30249 [35] and GSE80699 [36]. To compare genes responding to multiple stresses across species and to detect the patterns of gene trait association with drought phenotypes that relied on tissue-specific differential gene expression, we also used maize drought expression dataset from GSE40070 [37]. To consolidate our analysis of multi-stress responses across species, we again used rice drought expression dataset from GSE57950 [38] and salt expression dataset from GSE73181 [39]. Drought and salt co-expressed genes were also identified in rice. Gene expression profiles for significantly expressed genes for all species based on drought and salt stresses are shown using a heat map and up-down regulated genes were visualized using a volcano plot for which a description of the P-value and fold-changes is shown in S1 Fig. Statistical significance was determined using parametric t-test (P-value < 0.01) to estimate the variance between subjects.

### Functional-annotation and GO enrichment

Analysis of GO functional annotation was conducted using agriGO [32] where gene ID were used as input and Blast2GO V4.0 [40], a standalone software that locally incorporates repository using MYSQL DB, for sequence based analysis. Gene Ontology assignment was used to classify the functions of the selected sequences. Basic Local Alignment Search Tool (Blast, Blastp [41]) was employed to detect the sequences that were mapped against the non-redundant NCBI protein database for the best Blast hits. The functional classification and distribution of genes into main and sub-GO categories was determined, while the GO-terms were demonstrated and summarized based on the GO functional annotation.

Gene association and functional enrichment network were determined based on the enrichment level of GO terms (p-value, False Discovery Rate (FDR) < 0.05). The GO-terms with p-value < 0.05 were considered significantly enriched for all the 3 domains, namely BP, CC and MF. Similarly, enriched genes (FDR < 0.05) which exhibited strong association with their respective plant attribute from TO, PO, GRO and EO were also determined. To visualize stress related GO-term associated genes, scatter plots for multidimensional scaling of semantic similarities and gene enrichment map for functional network of the genes were generated using default values in ReviGO [42] and Cytoscape V3.3.0 [43] respectively.

### Phylogenetic analysis

Sorghum protein sequences that represent 710 full length gene orthologs related with multiple stress responses in the 3 crop species (maize, rice and Arabidopsis) were retrieved from the Ensembl plant Compara multi-species database [44] for the purpose of multiple sequence alignment and phylogenetic analysis. These were subjected to a screening procedure to retain 450 genes (493 peptides) which were aligned using a multiple sequence alignment standalone tool ClustalW V2.1 [45]. In order to improve the overall sequence alignment, the iteration parameter was set to TREE to allow a retention of the resulting alignment if it was improved over the previous alignment at every iteration step. This was repeated to undertake progressive alignment for a number of rounds until the highest alignment score was met. The Maximum likelihood analysis alignment file obtained from ClustalW was used by ClustalW2 phylogeny (http://www.ebi.ac.uk/Tools/phylogeny/clustalw2_phylogeny/) to calculate a percentage sequence divergence based distance matrix and to generate a phylogenetic tree using the Nexus tree format and the neighbour-joining clustering method with the distance correction and the gaps exclusion parameters. The resulting tree file in the plain text format was uploaded, visualized and annotated in the iTOL [46].

### Identification of genes associated with Quantitative Trait Loci (QTLs)

In order to evaluate if some of the genes identified in this study for sorghum multi-stress responses are co-localized within any of the QTLs previously identified for stress tolerance, we examined and compared the genomic coordinates of the target genes with the QTLs known for drought tolerance [47–49]. The target genes that fall within the QTLs genomic regions were considered as potential candidates for association with these QTLs. In addition, maize and rice QTLs fasta sequences release were obtained from the Gramene database [50] and compared with the nucleotide sequences of our target genes identified for maize and rice stress tolerance that were retrieved from the Phytozome database [51] using BioMart [31]. The latter were used as the query sequences to align with a local Blast database created from the maize and rice QTL sequences using Blastn [41]. Best Blast hits were selected by extracting a unique target hit per query sequence based on bit score, e-value (1e-10), % identity and length of alignment.

## Results

### Semantic integration of data based on functional ontology

**Fig 2:**
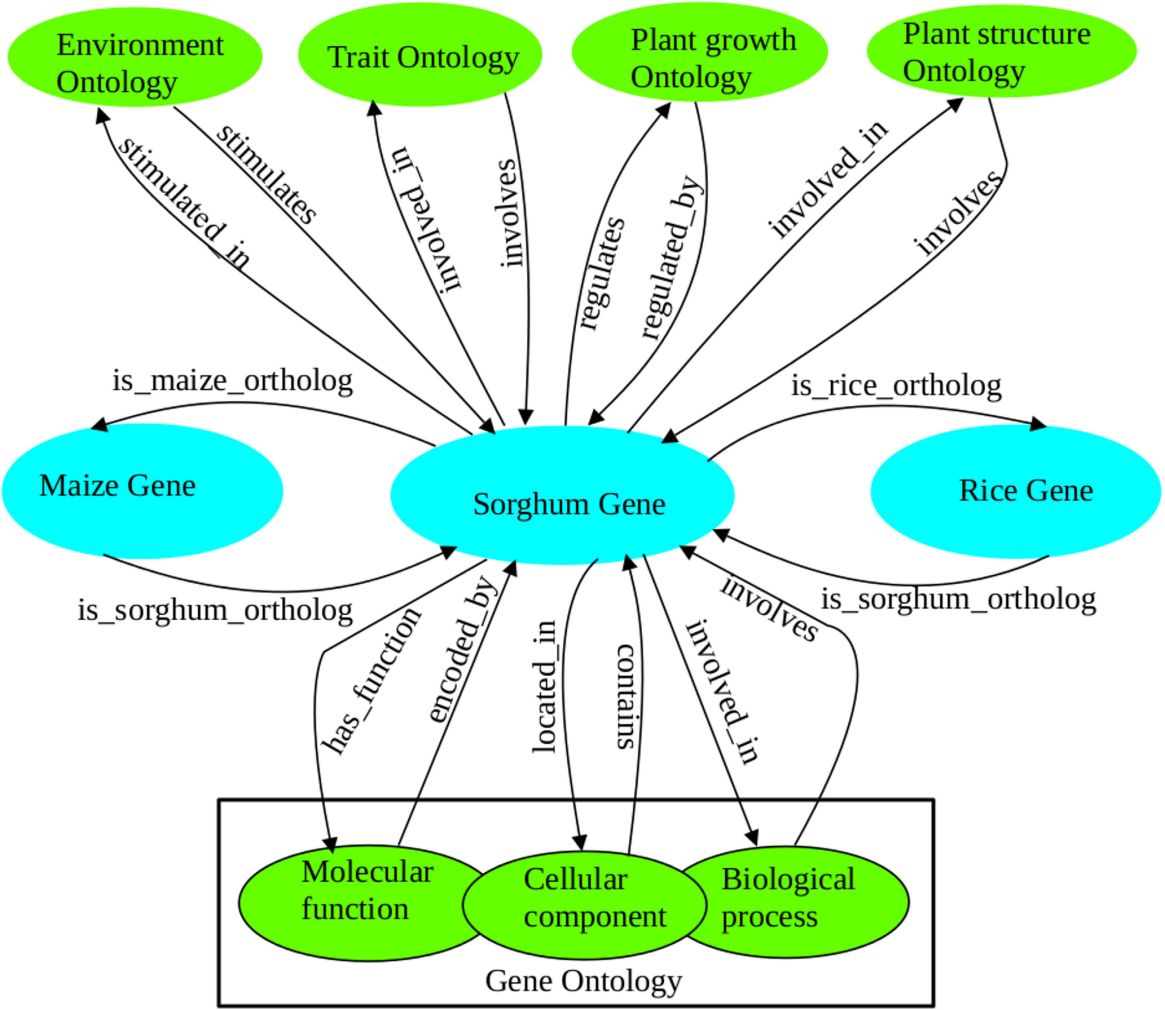
Ontology map for sorghum gene association to multiple drought related terms. The figure shows sorghum genes directly and transitively associated to multiple drought related terms based on functional ontologies. The information from EO, TO, GRO, PO and GO was used to investigate sorghum genes and orthologs in rice and maize associated with stress response. The map represents sorghum specific features for displaying class hierarchy against the ontologies under consideration and the orthologous genes from maize and rice. The hierarchical structure was designed to show multiparental relationships of sorghum genes with different ontology categories without including direct class hierarchy between maize or rice genes to the ontologies. This reveals the occurrence of multi-stress responsive sorghum specific genes and orthologous groups which are associated with GO cellular components for their localization. While the molecular functions and the biological processes of the sorghum specific genes and the orthologs are conserved, the ontology supports all these biological realities.

Semantic integration of information associated to sorghum and related model species stress perturbation resulted in a list of potential genes with direct and transitive relation to sorghum gene-trait association. This, however, also resulted in maize and rice specific gene-trait relationship. Where our query for relevant terms in the different ontologies yielded no existing information for sorghum, we opted to use transitive gene association to multiple traits through rice and maize orthologs. Therefore, transitive association of sorghum orthologs with drought related ontology terms was used for complementing sorghum related gene data to make sufficient association with multiple drought-related terms in several ontologies. We took advantage of the 19.6% direct and transitively associated sorghum orthologs of the identified 1709 candidate genes to construct the ontology mapping (Fig 2). This was employed to functionally validate the relevance of a total of 335 putative uncharacterized genes for drought response in sorghum (S1 Table).

### Gene expression profiling based on stress conditions

**Fig 3:**
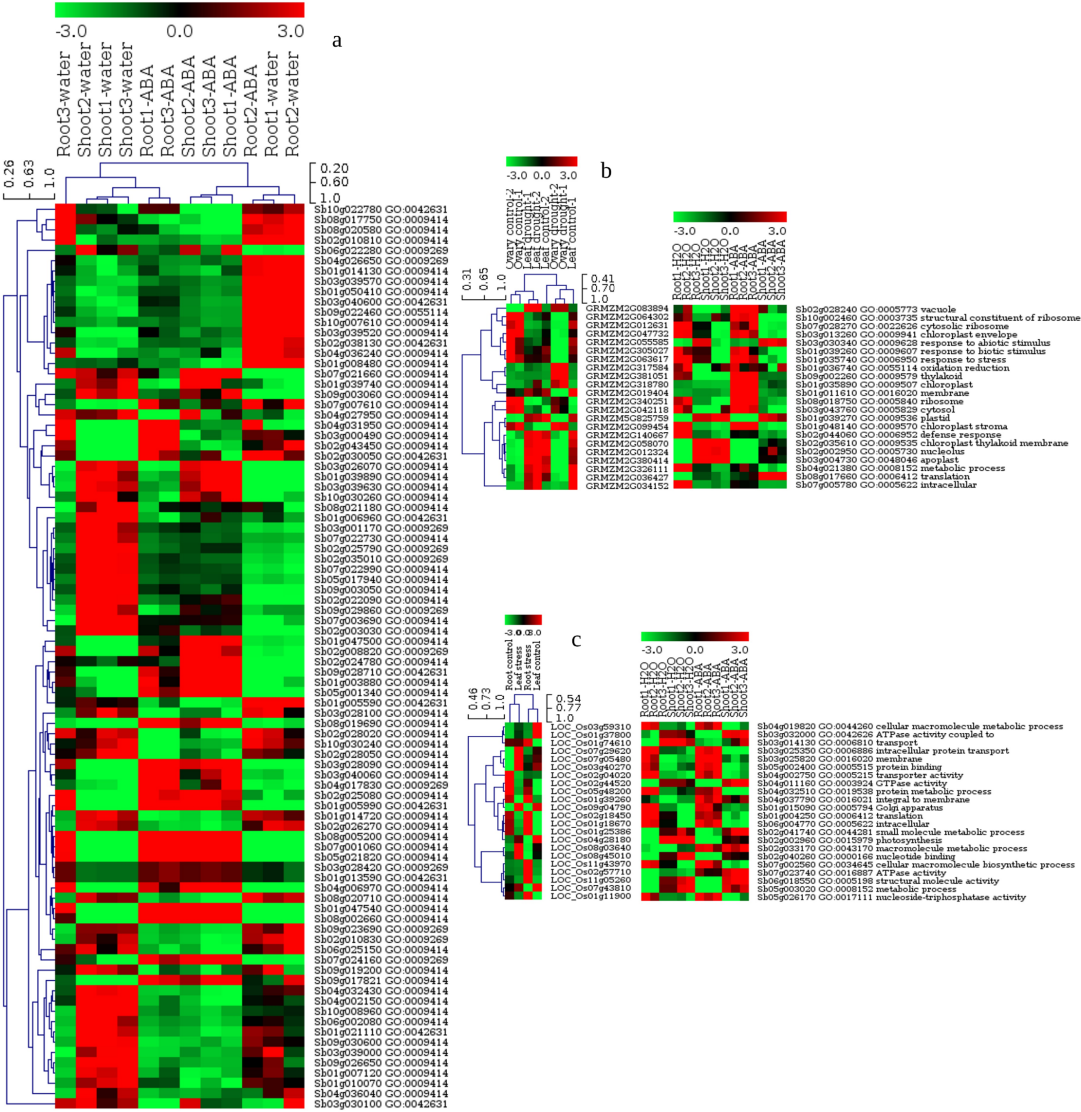
Heat map showing differential gene expression. This figure shows gene expression profiles based on sorghum drought stressed root and shoot tissues (a), the 22 most abundant GO terms enriched in maize leaf and ovary tissues under drought stress with the corresponding up-regulated maize genes and their respective sorghum orthologs expression patterns (b). Similarly, the figure shows the 22 common GO terms enriched in rice leaf and shoot under salt stress showing the corresponding up-regulated rice genes and their corresponding sorghum orthologs expression patterns (c). Sorghum orthologs expression patterns were added in (b) and (c) to show visual comparison of expression profiles for transitively associated genes between sorghum and maize and sorghum and rice separately. Parametric analysis of gene set enrichment was determined by the T-statistics based clustering frequency using MeV 4.48 [52], an R based software. The rows represent the genes (a), GO-terms and corresponding genes and orthologs and GO annotation (b) and (c), whereas the columns represent the biological samples. While the red color denotes the up-regulation, the green shows down-regulation of the genes in all the clustering panels. Hierarchical clustering, for instance show the patterns of expression in (a), by grouping the most up-regulated sorghum genes in the upper right corner, middle and lower left corner.

Expression data was integrated with information from functional ontologies that demonstrated a successful association of drought and related stress responsive genes with phenotypes. A total of 46 significantly up-regulated sorghum genes from GSE30249 [35] were shown to have strong correlation with drought tolerance based on the evaluation of tissue type contribution to the gene expression. The evaluation of treatment effect revealed 42 significantly up-regulated genes under drought condition for which association from all plant attributes was determined (Fig 3A and S2 Table). These results show that there was a higher percentage of gene representation in tissue-specific expression under stress condition than with drought stimulation irrespective of tissue type, in agreement with the previous report [35]. Analysis of differential expression that shows significantly up-regulated genes was also demonstrated using volcano plot both for the evaluation of tissue type gene expression profiles and the treatment effect on experimental samples (S1 Fig). To consolidate our result, we further evaluated sorghum gene expression profiling, using additional experimental dataset (GSE80699) generated under drought condition for 2 leaf genotypes [36]. A total of 347 highly expressed genes were identified, of which 201 were assigned to enriched drought related GO-terms, p-value, FDR < 0.01 (S2 Table). Among up-regulated sorghum genes that were identified under drought stress, Auxin-responsive protein IAA30, Heat shock protein 81–2 and 90, Late Embryogenesis Abundant proteins-like (LEA), putative senescence-associated protein and Zinc finger family putatively expressed protein are just a few to name (S2 Table). A combination of sorghum drought specific non-redundant set of genes identified from the two dataset is presented in S2 Table.

Based on maize expression dataset, GSE40070 [37], the pattern of expression profiling that was determined using parametric t-Test, p-value, FDR < 0.01 resulted in a total of 300 genes expressed under drought stress of which 200 were tissue specific. Evaluation of tissue distribution of significantly expressed genes showed more up-regulated genes in the reproductive stage than in the vegetative, more likely concomitant with the trend of expression pattern in [37]. Conversely, 125 up-regulated genes were obtained from the treatment based grouping, out of which 100 genes with best fold-changes were selected for functional gene enrichment and GO annotation analysis in combination with the result from tissue based grouping. This resulted in 156 genes annotated for enriched drought associated GO-terms (S3 Table). This result shows an additional finding of drought expressed, tissue specific genes compared to what Kakumanu and colleagues had previously identified and presented in their final list [37]. The heat map for hierarchical clustering of 22 most abundant enriched drought related GO-terms including GO IDs is shown depicting the expression patterns of the maize genes and the corresponding sorghum orthologs (Fig 3B).

Analysis of the rice drought expression dataset, GSE57950 [38] showed 284 significantly expressed genes, p-value < 0.05 (S4 Table). Similarly, rice salt expression dataset, GSE73181 [39], revealed 164 tissue specific genes and 161 genes regulated irrespective of the influence of tissue on their expression pattern. Among tissue specific genes, 97 were annotated for enriched salt specific GO-terms, while among genes expressed regardless of tissue type, 36 were assigned to salt related GO-terms, p-value, FDR < 0.01 (S4 Table). However, when evaluated for multi-stress responses in rice, 84 genes were shown to be co-expressed both under drought and salt stresses. We show the heat map for hierarchical clustering of 22 most abundant enriched GO-terms with the corresponding GO IDs for drought and salt co-expression demonstrating expression patterns of the rice genes and the corresponding sorghum orthologs (Fig 3C).

### Gene association across-environmental stresses: Cross-talk and specificity

**Fig 4:**
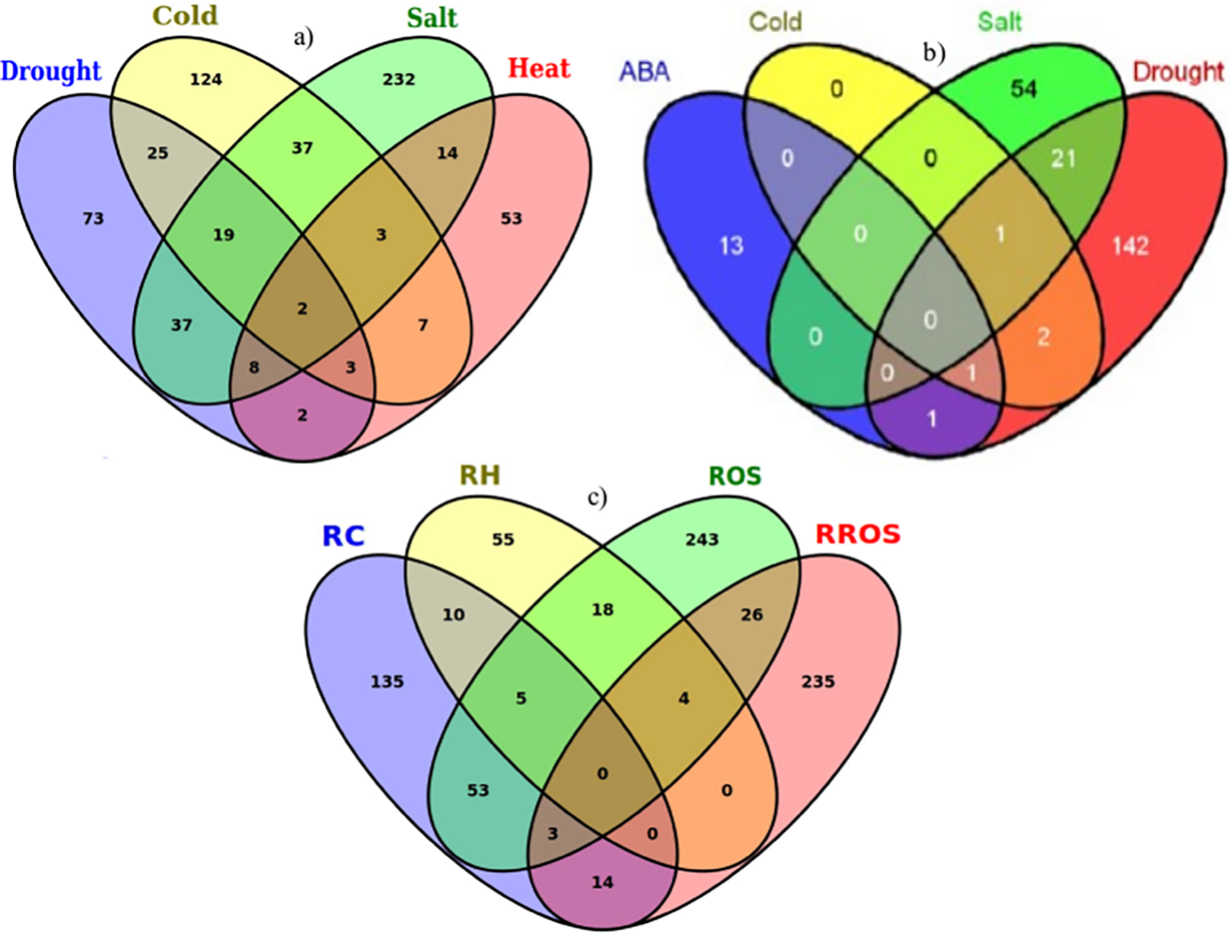
Venn diagram to show functional cross-talk and specificity of genes for drought tolerance and other stresses. Sorghum genes are shown in association with drought, salt, cold and heat stress related ontology terms of the biological process based on the datasets originated from Gramene database (a) and those in association with drought, ABA, cold and salt stresses based on sequence similarity search using expression dataset (b). Similarly, sorghum genes associated with ontology-terms of the biological process related to stress other than drought (salt, cold and heat and reactive oxygen species) based on data from Gramene database (c) are presented. The numbers displayed in the Venn diagram correspond to the number of genes. The superimposed regions of all circles show the number of genes shared in all the four species. The peripheral parts that don't overlap between circles show unique genes responsive to the respective stresses. **Key to legend:** RC—Response to cold; RH—Response to heat; ROS—Response to osmotic stress; RROS—Response to reactive oxygen species.

Sorghum genes association for functional cross-talk and specificity was investigated for drought tolerance and other stresses (Fig 4A, 4B and 4C). Among the 169 genes initially identified as drought responsive in sorghum based on the GO biological process, about 56% were shown to be responsive in multiple environmental stresses (Fig 4A and S5 Table). Among these multi-stress responsive genes, about 69% were salt responsive, whereas 51 and 15% were responsive to cold and heat respectively. Again, 22, 11 and 4% of the genes were shown to have dual function towards salt and cold; salt and heat and cold and heat responses respectively. Interestingly, 2% of the genes were shown to have universal responses to all the stresses under investigation (Fig 4C and S5 Table).

Based on the biological processes of the GO category, 1116 sorghum candidate genes were identified to respond to 5 different abiotic stresses, viz., drought (169), salt (352), cold (221), heat (92) and oxidative stress (282) (Fig 4, Table 1 and S5 Table). A diverse functional array of sorghum gene association is characterized by the over-expression of some specific genes for multiple traits. For instance, 2 peculiar genes (Sb03g026070 and Sb09g030600) were identified to be expressed in all the 4 stresses, namely drought, salt, cold and heat (Fig 4A). Furthermore, many other genes were shown to have common expression in 2 or more abiotic stresses. For example, 2 genes (Sb01g037090 and Sb02g043450) for drought, cold and heat, 2 other genes (Sb03g039820 and Sb09g022290) for drought, salt and oxidative stress, again 2 other genes (Sb01g003880 and Sb10g023010) for drought and heat and 3 more genes (Sb0010s007790, Sb01g031520 and Sb10g022780) for drought and oxidative stress were found to be commonly expressed (S5 Table).

**Table 1:** Description of the GO enrichment analysis with enrichment level of the GO-terms in decreasing order, the corresponding number of drought responsive genes involved and the associated traits in each GO-category.

GO-category	GO-term	GO-ID	# of genes	P-value	FDR	Traits
Biological process	response to water deprivation	GO:0009414	138	1.80E-039	8.90E-036	Drought stress tolerance
Biological process	response to cold	GO:0009409	138	7.40E-021	3.70E-018	Cold tolerance
Biological process	response to osmotic stress	GO:0006970	65	1.50E-012	2.50E-010	Osmotic stress tolerance
Biological process	response to salt stress	GO:0009651	93	1.80E-009	2.10E-007	Salt stress tolerance
Biological process	response to desiccation	GO:0009269	25	5.90E-008	4.70E-006	Drought stress tolerance
Biological process	response to oxidative stress	GO:0006979	95	9.00E-008	7.00E-006	Oxidative stress tolerance
Biological process	response to reactive oxygen species	GO:0000302	43	5.00E-007	40E-005	Oxidative stress tolerance
Biological process	oxidation reduction	GO:0055114	62	5.80E-007	3.80E-005	Drought stress tolerance
Biological process	response to heat	GO:0009408	56	3.30E-006	0.00019	Heat tolerance
Cellular component	plastid	GO:0009536	294	1.60E-015	9.20E-014	Drought stress tolerance
Cellular component	chloroplast	GO:0009507	257	6.50E-014	3.40E-012	Drought stress tolerance
Cellular component	chloroplast thylakoid	GO:0009534	96	2.90E-013	1.50E-011	Drought stress tolerance
Cellular component	thylakoid	GO:0009579	103	2.80E-012	1.20E-010	Drought stress tolerance
Cellular component	chloroplast stroma	GO:0009570	37	4.00E-008	1.20E-006	Drought stress tolerance
Molecular function	oxidoreductase activity	GO:0016491	285	4.80E-009	8.30E-007	Drought stress tolerance
Molecular function	protein binding	GO:0005515	676	3.90E-007	4.30E-005	Drought stress tolerance
Molecular function	water channel activity	GO:0015250	14	9.40E-005	0.0049	Drought stress tolerance

Similar results were also observed for the large number of genes interacting across environmental stresses. For example, 8 genes were shown to act commonly in 3 stresses: i) drought, cold and oxidative stress and ii) drought, heat and salt each (Fig 4A and 4C). Seventeen genes in drought and cold, 19 genes in drought, salt and cold and 35 other genes in drought and salt were commonly responsive (Fig 4A and S5 Table). The distribution and functional correlation of genes associated with abscisic acid (ABA), drought, salt and cold stresses as indicated in Fig 4B were basically dependent on the extent of sequence similarity. The pattern of functional association for genes that are purely drought responsive (Fig 4C) was depicted based on the enriched drought related GO-terms of the biological process. Stress specific expression of genes in all association was also shown (Fig 4A, 4B and 4C); for instance, 71, 232, 208, 120 and 53 genes were found to be uniquely specific to drought, oxidative stress, salt, cold and heat, respectively, as indicated in Fig 4A and S3 Fig.

### Comparative gene association across-species

**Fig 5:**
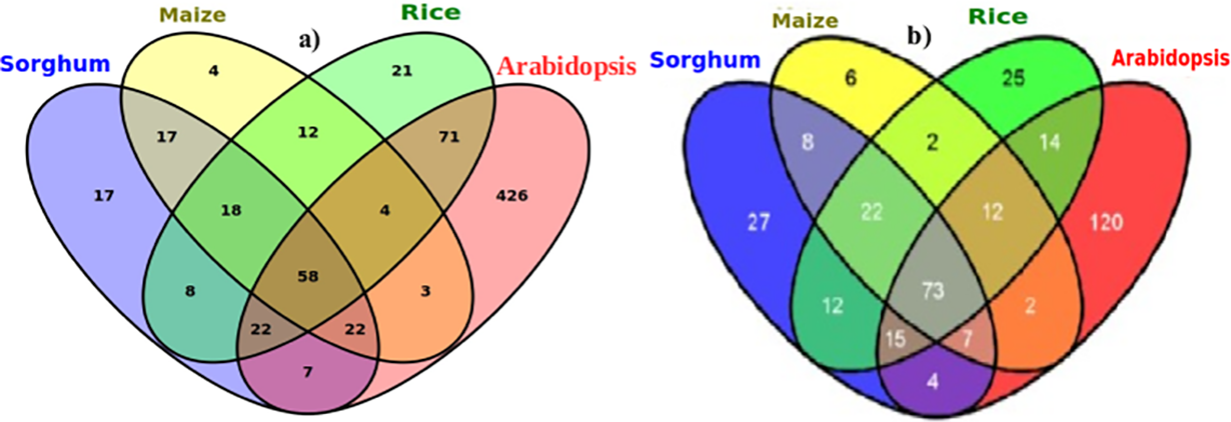
Venn diagram showing distribution of shared drought responsive genes among species and specific genes to sorghum. The figure shows a distribution of sorghum orthologous genes in the other 3 related species in association with drought related ontology terms based on existing data for known genes in Gramene database (a) and based on sequence similarity search using expression dataset (b). The numbers displayed in the Venn diagram correspond to the number of genes. Superimposed regions of all circles show the number of genes shared in all species under investigation. Overlapping regions between any 3 species indicate shared gene loci and functional conservation between the 3 of the 4 species while the shared regions between any 2 species involved show the shared gene loci and functional conservation in the 2 species. Parts that don't overlap between circles show unique drought responsive genes for each species.

Among a total of 168 sorghum drought responsive genes identified based on sequence alignment, 90% were found to exhibit drought tolerance across species without expressing any sorghum specific genes (Fig 5). Sorghum genes' functional correlation with orthologs in other species showed that 11% were shared with maize only, nearly 5% with rice only and 5% with Arabidopsis only. Again, 12% of sorghum genes were shared with maize and rice in common and 15% with rice and Arabidopsis. Moreover, 34% of the total sorghum genes were commonly shared by all 3 species. This shows the presence of species specific and shared gene loci and probably functional conservation in closely and distantly related species of grass families. The total number of drought responsive genes represented in sorghum, maize, rice, and Arabidopsis were 335, 138, 214 and 613, respectively (Fig 5A and 5B; S1 Table). Such a representation of drought responsive genes in each species in this data was based on the relevant drought related terms in the EO, TO, PO, GRO and the GO (Table 2, S6 Table and Fig 5A). However, it is important to note that potential genes for drought tolerance in sorghum having shared functionality with closely related species were identified based on the putative functions of their orthologs in all related species using Blast algorithm and the Ensembl BioMart as described in the method. The identification of a relatively larger number of both shared drought responsive genes among all species and that of sorghum specific based on sequence similarity search using expression data (Fig 5B) than based on querying known genes in Gramene database (Fig 5A) suggests the presence of new biological information content in the expression dataset.

**Table 2:** Summarized description of functional association of genes with various drought related ontology terms identified at different stages based on step-wise screening procedure.

Ontologies	Ontology terms	Ontology accessions	Identified genes	Merged and screened genes
Gene Ontology	Biological process	GO:0009414	167	126
	Cellular component	GO:0005575	148	
	Molecular function	GO:0003674	133	
Trait Ontology	Drought tolerance	GO:0009414, GO:0009819	150	296
	Chlorophyll content	TO:0000495	12	
	Stay green trait	TO:0002712	2	
	Biochemical trait	TO:0000277	2	
	Leaf senescence	TO:0000249, GO:0010150	132	
	Growth & development trait	TO:0000357	2	
Environment Ontology	Drought environment	EO:0007404	1165	1681
	Sodium chloride regimen	EO:0007048	1193	
	Salt regimen	EO:0007185	398	
	Watering regimen	EO:0007383	2406	
	Cold temperature regimen	EO:0007174	1372	
Plant structure ontology	Inflorescence	PO:0009049	10200	98
	Tassel inflorescence	PO:0020126	32	
Growth Ontology	Reproductive stage	GRO:0007140	2803	712
	Seedling stage	GRO:0007047	9088	
	Booting stage	GRO:0007148	286	
	Early-booting stage	GRO:0007149	1949	
	Late-booting stage	GRO:0007150	1	
	Flowering Stage	GRO:0007151	6497	
	Heading stage	GRO:0007044	6454	
**Total**	**23**	**23**	**11,987 (unique)**	**2,224 (unique)**

### Phylogenetic relationship

**Fig 6:**
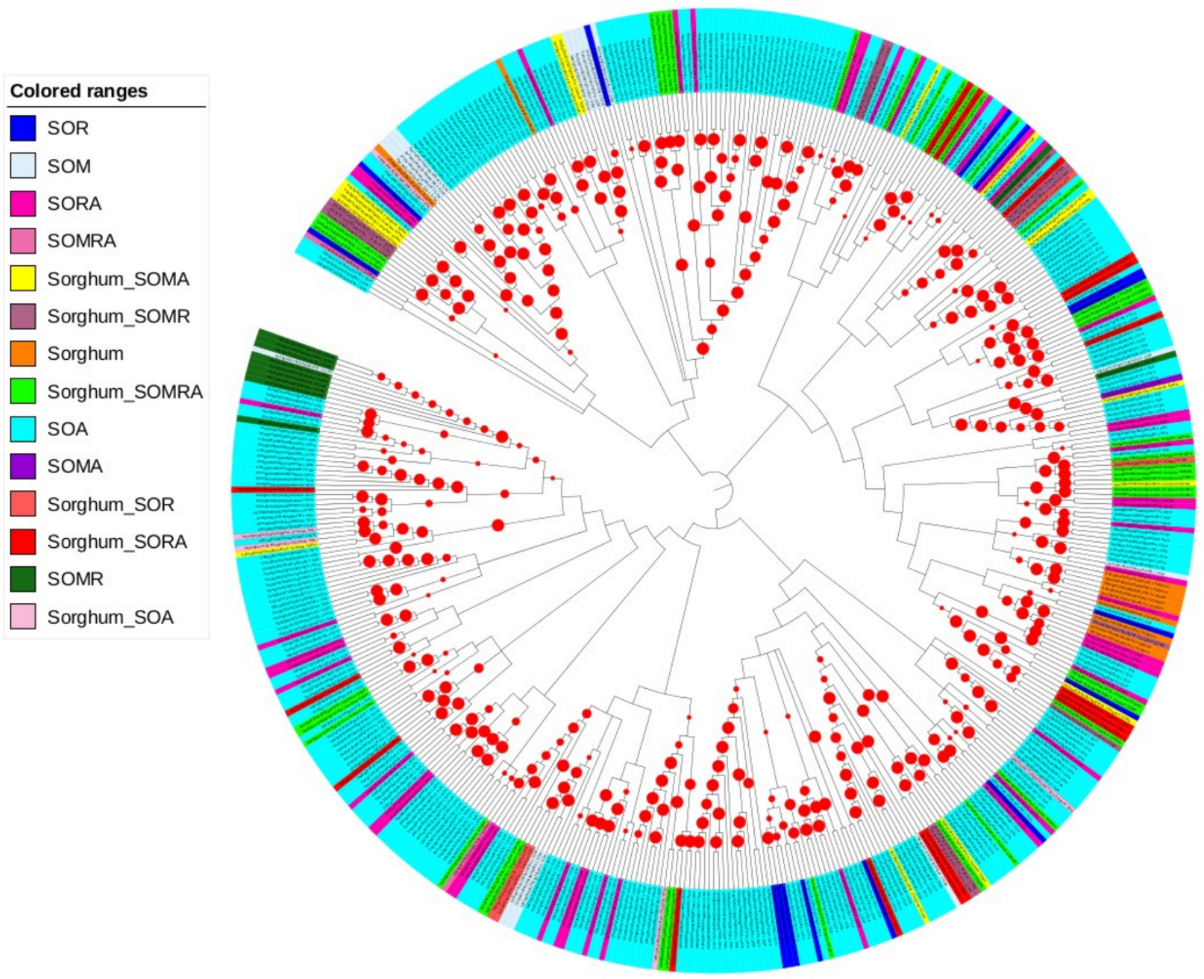
Circular representation of the polar formatted phylogenetic tree of the sorghum specific and orthologous genes. Group of genes were color-coded by orthology group identified for drought response in the other species evolutionarily related to sorghum. The tree represents labels that were aligned with default leaf sorting. Branches represent evolutionarily related ortholog clades. Branch lengths for which 'ignored' setting was adjusted were represented each by the numbers in decimal and the bootstrap values in absolute numbers (S10 Table). The tree was reconstructed after removing the gaps using a bootstrap support of the 1,000 replicates to show the frequency of each internal node, clades in the tree. The red circular bootstrap symbol was used to indicate the bootstrap supported clades based on the values within the range of 100 (small dot)– 1000 (large dot) iterative replicates, where more than 75% of the clades showed the bootstrap above the commonly known threshold value (70%). The clades with the bootstrap values less than 5% were removed from the tree. The values for the robust bootstrap support were given in S10 Table. **Key to legend for the colored ranges:** SOA, Sorghum orthologs in Arabidopsis; SOM, sorghum orthologs in maize; SOMA, shared sorghum orthologs in maize and Arabidopsis; SOMR, shared sorghum orthologs in maize and rice; SOMRA, shared sorghum orthologs in maize, rice and Arabidopsis; SOR, sorghum orthologs in rice; SORA, shared sorghum orthologs in rice and Aabidopsis; Sorghum, sorghum specific genes; Sorghum_SOA, shared sorghum specific and sorghum orthologs in Arabidopsis; Sorghum_SOMA, shared sorghum specific and sorghum orthologs in maize and Arabidopsis; Sorghum_SOMR, shared sorghum specific and sorghum orthologs in maize and rice; Sorghum_SOMRA, shared sorghum specific and sorghum orthologs in maize, rice and Arabidopsis; Sorghum_SOR, shared sorghum specific and sorghum orthologs in rice; Sorghum_SORA, shared sorghum specific and sorghum orthologs in rice and Arabidopsis.

Phylogenetic tree of the sorghum specific and orthologous genes identified for drought response in the other 3 evolutionarily related species to sorghum is displayed in Fig 6. The tree represents evolutionarily related ortholog clades, with branch lengths showing the amount of genetic changes between the clades. Distinct classes of evolutionarily related genes were found to be conserved across species, while relatively few sorghum specific genes were shown to exist. This comparative sequence evolutionary pattern across species for drought response was depicted based on the protein sequences identified in sorghum and its close relative species (S7 Table). The number of protein sequences evaluated for functional conservation across species was dependent on the availability of the protein sequences queried per species in the existing database. For instance, a ClustalW phylogenetic tree of these proteins revealed 297 genes functionally conserved between sorghum and Arabidopsis (SOA) among initially identified (613) and combined (782) orthologs (S8 and S9 Tables). Of the initially identified sorghum orthologs in maize (SOM, 138 genes), 4.5% account for 3.3% of the total conserved genes, whereas 8% of the 214 initially identified and 1% of the combined sorghum orthologs in rice (SOR) altogether accounted for 4.3% of the total conserved genes.

When viewed in terms of the number of sorghum orthologs conserved among more than 2 species, we found 44 (9%) drought responsive genes in all the species, 63 genes (13%) among sorghum, rice and Arabidopsis, and 22 genes (4.5%) among sorghum, maize and rice. Furthermore, 19 genes (4%) were shown to be shared among sorghum, maize and Arabidopsis. On the other hand, 5.3% of the 169 sorghum genes that were initially identified as drought responsive remained sorghum-specific, while the rest were evolutionarily conserved in 1 or more other species (Figs 5 and 6). These cross species conserved orthologs were classified into 14 subclasses based on the number and the type of species in which the orthologs were commonly occurring. We show shared orthologous genes and the type of species in which these genes are conserved (Fig 6 and S9 Table).

### Functional-annotation and enrichment of plant ontology terms

**Fig 7:**
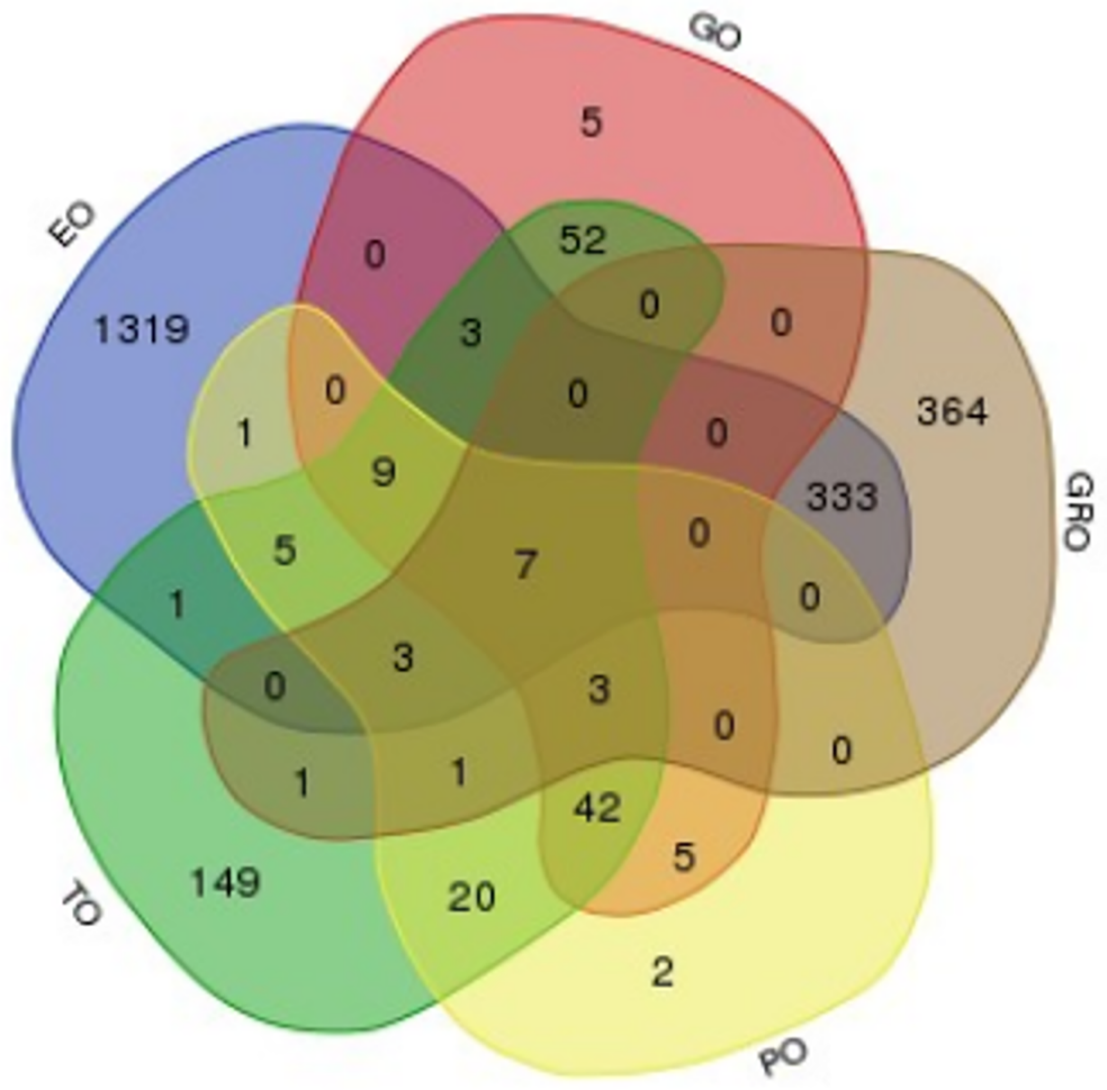
Venn diagram showing the sorghum orthologous genes identified in different plant ontology categories. The diagram represents the distribution of gene association related to the five drought-associated plant ontology terms. The numbers in the overlapping portions represent the number of gene contributions shared by 1 or more ontology categories with the genes positioned in the center represented by all ontologies, while those shown on the peripheral portion represent the number of genes specific to each respective ontology category.

Based on the association of genes with drought related GO terms, 167, 148, 133 significantly enriched genes (Table 1; p-value, FDR < 0.05) were identified for all the 3 domain namely BP, CC and MF respectively. This was further filtered to 126 non-redundant genes supported by all GO-domains. Similarly, using the same method, 296, 1681, 98 and 712 enriched genes (p-value, FDR < 0.05) were filtered from TO, EO, PO and GRO respectively, which were shown to have strong association to the plant attributes (Fig 7; Table 2; S6 and S11 Tables). The combination of these makes a total of 2224 filtered non-redundant genes which were further screened down to 2118 enriched transcripts or 1820 genes (S2 Fig and S6 Table). The distribution of these sorghum orthologs based on ontology categories is depicted using a Venn diagram, where the EO was shown to contribute the highest proportion (72%), followed by GRO (30.6%), TO (12.7%), GO (5.4%) and PO (4.2%) with 7 genes shared by all ontologies (Fig 7).

Gene ontology assignments were employed to functionally group the genes. Based on the Blast2GO analysis of sequence homology, 2357 annotated sequences that had received Blast hits from the non-redundant NCBI protein database were classified into 28 functional groups under the main categories of the GO classification (S3 Fig and S11 Table). In the BP, the metabolic process, single-organism process, response to stimulus, biological regulation and regulation of biological process were noted to be dominant to which 227 genes (9.6%), 197 genes (8.4%), 118 genes (5.0%), 106 genes (4.5%), 100 genes (4.2%) were classified respectively. The rest functional groups of the BP contributed to the classification of a total of 783 genes (33%). In the CC of the GO category, the predominant categories were cell and cell part each accounted for the functional classification of 230 genes (9.8%). While the organelle, membrane and membrane part contributed for the classification of 172 genes (7.3%), 110 (4.7%) and 83 genes (3.5%) respectively, the rest functional groups of the CC accounted for the total of 101 genes (4.3%). On the other hand, in the functional category of MF, the binding and catalytic activity each predominantly accounted for the classification of 184 genes (7.8%) and 171 genes (7.3%) respectively. While the highest percentage of genes from binding, cell and cell part and metabolic process was noted, only few genes were detected from the category of molecular transducer activity, symplast and positive regulation of biological process in the main GO categories of the MF, CC and BP respectively. A graphical representation of significantly enriched GO-terms assigned to the identified genes that demonstrated strong association with drought-responses was demonstrated using scatter plots and GO annotation and classification (S3 Fig).

### Gene functional enrichment network

**Fig 8:**
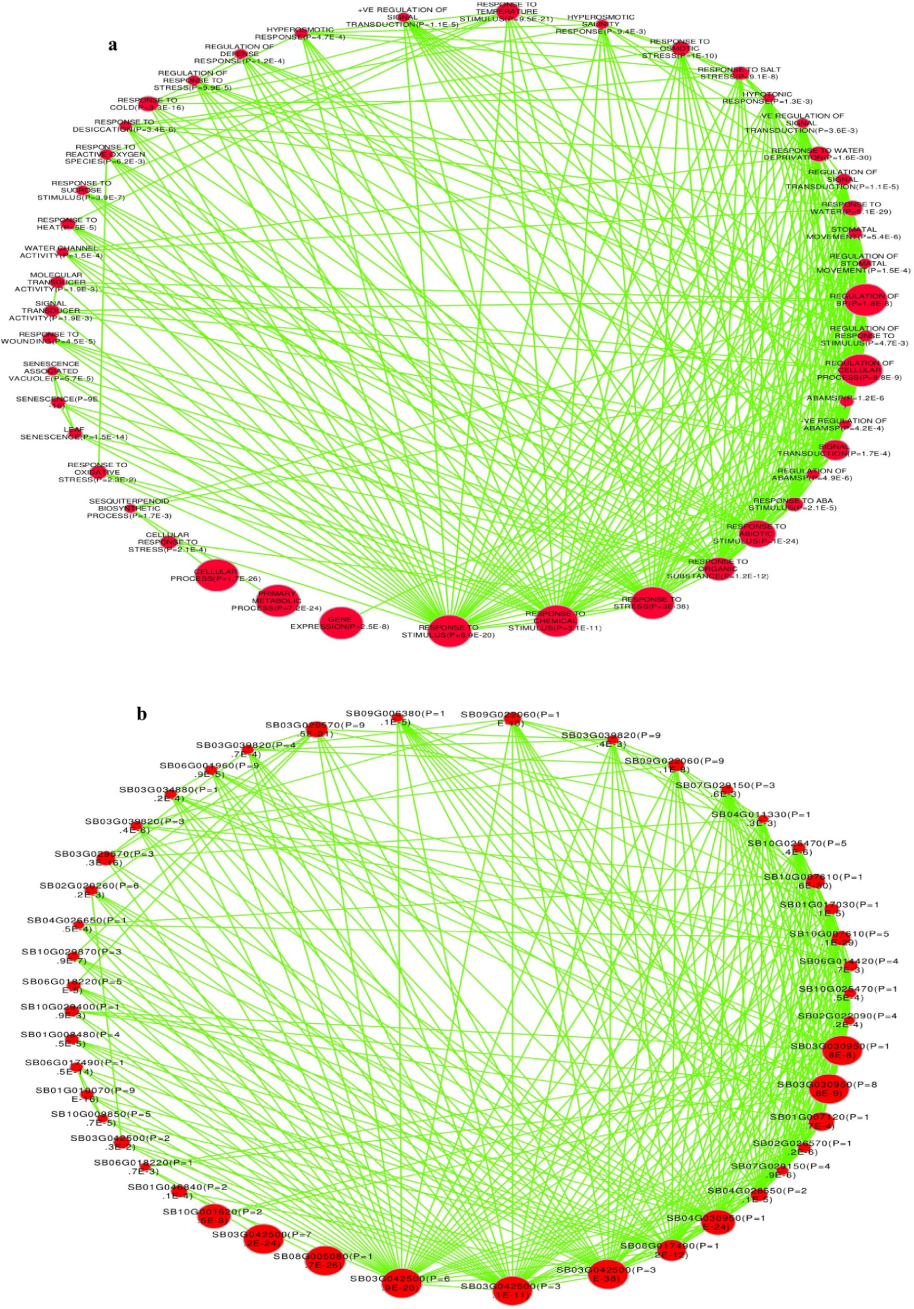
Enrichment network map for selected sorghum drought responsive genes. Gene enrichment maps for selected 50 sorghum drought associated enriched GO-terms (a) and the corresponding genes (b) depict the biological networks of the genes that are involved in the regulation of cross talk in response to multiple stresses. Nodes denote a group of genes (gene-sets) or group of GO-terms and edges represent GO defined relations. The threshold level of the enrichment significance determines the appearance of the group of genes on the enrichment network map. The intensity of the node represents the level of significance of the enrichment and the size of the node correlates with the size of significantly enriched gene set that overlaps or makes the group of up-regulated enrichment. The p-value is included in the label of the nodes to indicate the level of enrichment significance. The network explains the corresponding gene function defined by the enriched GO-terms in the particular GO-category. The position of the nodes for the enriched gene set is not necessarily correlated with the position of the corresponding enriched set of GO-terms. The color usage for the node and edge is an arbitrary selection for proper contrast.

Biological networks of gene association for which enriched GO-terms exist can be shown by using interactive biological networks [43] based on all deterministic factors attributed to the 3 GO-categories [53]. The gene enrichment network maps for selected 50 functionally enriched drought related GO-terms and their corresponding genes (P-value, FDR < 0.05) are shown in Fig 8A and 8B respectively. While mostly difficult to precisely interpret the functional networks and interactions of the genes, we opted to summarize the results as indicated in this article. The responses to stress, stimulus, chemical and abiotic stimulus and response to organic substances were shown to take the leading position with high significance of enrichment. The network denoted by the first 3 enriched GO-terms indicated above corresponds with the gene sets represented by 'Sb03g042500', the gene that also partly regulate the functional network of the primary metabolic process and the response to oxidative stress. Similarly the network represented by the last 2 enriched GO-terms corresponds with the gene sets represented by 'Sb04g030950' and 'Sb06g017490' respectively where the latter also coordinates the functional network and genetic interaction for leaf senescence. Moreover, the functional regulation of cellular and biological processes were shown to be controlled by the common set of genes represented by 'Sb03g030950', a gene that is also responsible for the functional network of response to abiotic stimulus. The biological network of the signal transduction was noted to be regulated by the set of genes represented by the gene, 'Sb01g007120' (Fig 8A and 8B; S12, S13 and S14 Tables).

It was also noted that the functional network of response to cold and temperature stimulus; response to heat and sesquiterpenoid biosynthetic process; response to water and water deprivation and response to osmotic and salt stresses were shown to be regulated by the interaction of set of genes each represented by Sb03g029570, Sb06g018220, Sb10g007610 and Sb09g022060 respectively. Likewise, the functional network of the hyperosmotic response, hyperosmotic salinity response and response to desiccation were noted to be controlled by the group of genes represented by Sb03g039820. The network for the negative regulation of signal transduction and regulation of abscisic acid mediated signaling pathway was however controlled by a common gene set denoted by Sb07g029150. Again, functional network for stomatal movement and regulation and molecular and signal transducer activity was detected to be controlled by gene sets each represented by Sb10g025470 and Sb10g029400, respectively.

### Resistance from whole-plant to individual level components

This study identified multiple individual level components that represent various drought response mechanisms specifically related to tissue type and developmental stage. A total of 669 genes which were manifested via osmotic adjustment (OA, 52.6%), antioxidant capacity with response to oxidative stress (42%) and desiccation tolerance (5.4%) were identified for DT in general. In addition, 19 genes with cellular responses to water deprivation and 126 genes with physical response to water deprivation were identified as DA category. Furthermore, a remarkably high number of genes (2442) responsible for DE were also identified for which relevant genes associated with early booting and a gene “Sb03g003110”, a rice ortholog (BGIOSGA002217) with late booting character were filtered (S15 Table).

### Identification of target genes associated with QTLs

This study has identified a total of 272 target genes which were associated with QTLs related to different traits including 62% of gene association from sorghum, 7.8% from maize and 30.2% from rice genes. One hundred and sixty-nine sorghum target genes initially identified for multi-stress tolerance were found to be associated with QTLs responsible for various traits. This includes 37 (21.9%) genes associated with drought adaption [47], 84 (49.7%) genes responsible for grain yield, flowering time, and stay-green traits [48] and 28.4% of the genes associated with seed dormancy [49] (Table 3; S16 Table). This study also identified 21 target genes in maize that are associated with drought QTLs of different agronomic purposes using sequence alignments based on Gramene QTL release [50]. The best hits were selected based on the % identity, e-value, bit-score. QTLs for abiotic stress tolerance (33.3%), biochemical (33.3%), developmental (4.8%), quality (4.8%) and yield (23.8%) traits were included. The abiotic stress tolerance QTLs were noted to play the main role in turgor pressure, stomatal conductance and abscisic acid concentration in plants, whereas the QTLs for the biochemical traits control the total soluble sugar content, ADP glucose pyrophosphorylase activity, peroxidase-71 and sucrose contents (S17 Table). In addition, other QTLs regulating female floral development, tenderness quality, ear number and seed weight were also identified in maize. Molecular markers linked to these QTLs and corresponding QTLs for the target maize genes are listed in S17 Table.

**Table 3:** Description of sorghum target genes associated with QTLs known for drought tolerance.

QTL id [Ref]	Traits	Location	Start	End	Co-localized genes identified in this study	
					Representative (location)	Total
QYLD1.2 [48]	grain yield	Chr 1	11145830	12704841	Sb01g012195 (11188494–11188718)	1
QYLD1.3 [48]	grain yield	Chr 1	11203256	21602500	Sb01g012230 (11244033–11246659)	8
QSDW1 [47]	shoot dry weight	Chr 1	59861427	64432960	Sb01g036220 (59863444–59868128)	6
QGI-1 [49]	seed dormancy	Chr 1	52962744	55721536	Sb01g030510 (52963286–52964344)	13
QYLD2.1 [48]	grain yield	Chr 2	63084956	63712593	Sb02g027900 (63173883–3174687)	6
QRDW1_2 [47]	root dry weight	Chr 2	71995008	77001005	Sb02g037700 (71999473–72002734)	8
qGI-3 [49]	seed dormancy	Chr 3	68132731	72423918	Sb03g040510 (68140707–68142336)	9
QSPAD4.1 [48]	stay-green	Chr 4	6803028	10119054	Sb04g006830 (6853756–6858575)	26
QYLD4.1 [48]	grain yield	Chr 4	45937548	62339532	Sb04g019670 (45942011–45943013)	10
qFv/Fm4.1 [48]	stay-green	Chr 4	64497114	65560043	Sb04g034665 (64501979–4504340)	18
qGI-4 [49]	seed dormancy	Chr 4	57546281	58537697	Sb04g027660 (57562353–57564270)	8
QRA1_5 [47]	nodal root angle	Chr 5	13413924	45779999	Sb05g007450 (13463573–13469204)	6
qFT6.1 [48]	flowering time	Chr 6	1402697	40763291	Sb06g001033 (1479485–1483300)	7
QYLD6.1 [48]	grain yield	Chr 6	50360463	52945337	Sb06g020970 (50361776–0364655)	3
qGI-6 [49]	seed dormancy	Chr 6	54128269	59786660	Sb06g025130 (54128281–54131608)	8
qGI-7 [49]	seed dormancy	Chr 7	59065206	60579009	Sb07g024070 (59081442–59086488)	7
QRA1_8 [47]	nodal root angle	Chr 8	8067699	41591844	Sb08g005781 (8160471–8161062)	3
QTLA1_8 [47]	total leaf area	Chr 8	47817803	48269890	Sb08g017820 (47829763–47830809)	3
QRDW1_8 [47]	root dry weight	Chr 8	48269890	50970340	Sb08g018270 (48273154–8279810)	4
qFT9.1 [48]	flowering time	Chr 9	4719436	7580762	Sb09g004180 (4910985–4919916)	5
qGI-9 [49]	seed dormancy	Chr 9	57746020	58246041	Sb09g028980 (57747092–57747957)	3
QRA1_10 [48]	nodal root angle	Chr 10	57494967	58573866	Sb10g027700 (57507083–57508898)	7

Furthermore, 82 target salt responsive genes that are associated with QTLs controlling different traits in rice were identified. The QTLs and the associated genes were subdivided into different categories based on the type of traits they control including abiotic stress tolerance (12%), anatomical (9.8%) and biochemical traits (12%), sterility or fertility (12.6%), yield (50%) and quality characters (1.2%). Two types of markers (RFLP, 56% and SSR, 44%) were identified to be genetically linked to the QTLs. The detailed description including QTLs and the corresponding marker IDs and species from which the markers originate is provided in S18 Table.

## Discussion

Advancing plant adaptation and responses to multiple individual or combined stresses is a vital means to improve crop productivity under a changing but unforseeably complex conditions. However, understanding the genetic basis of complex traits in plants remained challenging due to complexity in the stage and development specific physio-biochemical processes at cellular and whole-plant level [54]. Recent advances in molecular studies have shown that this challenge is tractable and within reach of functional genomics [55] and association studies [56]. Identification of genes associated with multiple stress responses and their functional conservation across species by and large, was successfully demonstrated in the current study, generating target genes linked to known QTLs for complex stress tolerance using an integrated, efficient and straight-forward approach. This study reports a multi-environmental stress tolerant genes, which were previously ascribed only as hypothetical proteins in sorghum and other model crop species revealing regulatory role of major genes involved in cross-talk and specific responses to broad range of stresses.

Multiple responses of genes across environmental stresses is the genetic foundation of plant adaptation to environmental heterogeneity. Most of the genes identified for sorghum drought response were shown to respond under several stress conditions suggesting that many of these genes are involved in the regulatory network for controlling pathways that cross-talk in multiple responses. More than 50% of these genes were found to be responsible for the defense and tolerance responses in multiple environmental stresses of which the majority were shown to be co-expressed in drought, salt and cold stresses, however, a good number of genes were also shown to be co-expressed under heat and oxidative stresses. The over and co-expression of these genes in two or more individually or simultaneously occurring stresses suggests their active involvement in a shared but complex multifaceted biological and cellular metabolic processes that allow cross-talk between multiple biochemical pathways in response to multiple stresses.

Comparison of this study with the previous investigations that employed integrative data analysis methods revealed the significance of the present approach in finding target genes for multiple stress tolerance across species. For instance, Makita et al. [57], using experimental and public dataset depicted expression profiles for genes that show co-expression and co-regulation. Another study employed different type of integrative approach via transcriptome analysis pipeline to process RNA-seq data and to ultimately produce co-expression networks along with functional and comparative genomics data analyses [58]. These studies are well in agreement with our approach in identifying co-expression of genes. The present approach managed to identify target genes that are simultaneously expressed in stress combinations to enable improvement of multiple stress tolerance in sorghum and other related model crops. In addition, earlier investigation used integrated functional annotation of genes to provide information on genes and orthologous relationships of sorghum with other species [59]. Furthermore, an integrative analysis system for plant systems biology was employed to integrate and analyse gene expression and metabolite profile datasets to provide biological and functional information using biochemical pathways and gene ontology terms [60]. All these studies are sufficiently concomitant with the present study signifying the importance of integrative data analysis approach to mine genes that are co-expressed and involved in multiple stress tolerance.

To this effect, multi-environmental expression of genes representing quantitative expression dynamics under varying stress conditions [25,61] provides an impression of how genes might be regulated in the plant pathways during simultaneous exposure to different stresses. In this regard, physio-biochemical and molecular mechanistic function of a gene, across environmental heterogeneity, where respective stresses are prevailing such as observed in the present study, may represent a fundamental element employed in multiple stress tolerance.

The resolution of the whole-plant resistance into individual interrelated components was made possible through identification of functionally enriched drought expressed genes which were associated to predetermined stress relevant ontology terms. Drought resistance can be broken down into 3 main component parts [62] such as DT, DA and DE that were all identified in this study. Drought tolerance investigation contributed to the large number of genes characterized by tolerance to osmotic stress, oxidative stress and desiccation which were probably associated with sorghum morphological and physio-biochemical responses [63] and with the regulation of accumulation and translation of assimilates and maintenance of cell wall elasticity [64]. While DA enhances plant water uptake minimizing evapotranspiration, DE, spanning a short life cycle or developmental plasticity of the plant, allows the plant to by-pass the window of stress. The finding of these individual level components confirms that our results align with the previous finding [62] and that most of the identified genes satisfied the drought resistance criteria, suggesting that our strategy represents a more characteristically holistic and promising for dissecting the complex polygenic traits into particular elements of plant DR.

A highly diverse genetic basis and rich functionality of cereal crops such as sorghum that engage gene association with important and complex traits, provides a foundation for adaptation to adverse environments. The initially identified 1681 genes (75.5%), based on drought stress related environmental regimes that were commonly enriched by all EO terms, confirms that sorghum is one of the few crops with potential sources of improved multi-stress tolerance. Our analysis shows that this approach is effective in examining an interoperability of plant ontologies which are not functionally overlapping but pointing to the interrelationship of the plant traits with all other plant attributes including the plant environmental regimes. This suggests that the extent of plant adaptability, survival and productivity are empirically associated to the genetic make up of the plant itself and the conditions that influence the optimal performance of all attributes which include traits such as chlorophyll content, stomatal closure, morphological and anatomical structural fitness as well as early or late maturity. The genes identified in this study were shown to be involved in determining cross-species phenotypic patterns under multiple stress conditions and are probably associated with their biological functioning.

Analysis of cross-species gene association among the 4 related species, suggests the existence of homologous groups, that descend from a common ancestral gene pool [65]. This entails an evolutionary proximity of sorghum to the other 3 species and the conservation of specific genomic regions across species with certain level of similarity in functional association to drought tolerance. Orthologs, unlike paralogs which evolve to functional diversification [66], typically occupy the same functional niche in different organisms [67]. While orthology is related to conserved structural elements, one orthologous group often contains different functions [68] though sequence similarity alone may not represent a functional group. The presence of 10% sorghum specific genes implies a uniqueness of sorghum crop compared to the other species in this study suggesting its distinct position in phylogenetic order and the probable evolution of new functional genes as a consequence of long term adaptation. The presence of such unique genome encoded genes which are structurally and functionally, however, preferentially evolved, have developed sorghum-specific plasticity in response to changes in environmental conditions such as drought and related stresses. This further demonstrates the key role of associations between sorghum genes and drought phenotypes for their orthologous counterparts as a means for deciphering genetic dissection of complex drought tolerance.

The phylogenetic tree of life provides insight into evolution and functions of different orthologous clades of genes in the sorghum and the other species under investigation. The lower values of the branch lengths may indicate the minimal genetic changes which may vary over time that have undergone during evolution, suggesting the probable structural and functional conservation of the orthologous groups across species or at least a gene duplication event that might have occurred much earlier or just before sorghum diverged from the respective ancestor. Depending on the proximity of the species evolutionary relationship, not only the different subclasses were grouped in the same or different ortholog clades but also, orthologs of the same subclass were grouped in the same or different clades. This is because, orthology, in most situations, does not necessarily represent orthologous genes with the most similar sequences or structures and conversely, most similar genes to each other in compared sequences might not be orthologous [69].

Functional ontology has been instrumental for genetic deciphering of complex drought tolerance through semantic knowledge [70]. A semantic integration of sorghum perturbation based ontology mapping which was also related to transitive association of sorghum orthologs with drought related ontology terms is an implication of potential candidate genes for drought tolerance. In the current analysis, of the total genes that expressed association across all ontology terms, at least 50% had transitive association. This is largely because, gene ontology enables annotation of homologous gene and protein sequences across organisms based on shared biology and the association of genes to the respective nodes within an ontology [17]. Semantic knowledge based ontology mapping not only implicates the functional similarity of sorghum genes with orthologs from closely related species particularly maize and rice, but also suggests the conservation of gene functions between these species.

Importantly, the use of expression data to investigate cross-species gene association with multiple stress phenotypes was demonstrated. A number of previous studies have used different approaches to utilize expression data in combination with text information from several areas but not limited to quantitative genetics [71]; molecular breeding [72] and biomedical research [73]. Integration of expression data with functional ontology based information successfully identified the association of relevant genes related to stress tolerance with phenotypes in sorghum and other model species. Multivariate analysis provided a significant array of genes associated with drought tolerance with or without tissue specificity. It was noted that, among the genes tested for significant expression, over 50% showed strong association with drought response in sorghum and maize and drought and salinity tolerance in rice. This not only shows the significance of expression profiling in segregating genes based on their attributed association but also suggests its role in complementing other strategies in the study of plant stress tolerance.

The finding of 272 multiple stress responsive tissue expressed target genes associated with known QTLs that regulate complex stress tolerance not only signifies the importance of integrated approach in targeting co-localized regions that affect these traits in the respective plant genome but also in providing information towards understanding the mechanisms behind shared and unique responses to multiple individual or stress combinations. The findings also revealed the significance of this study on crop improvement and productivity, because QTL co-localization is an important approach of identifying traits for stress tolerance and yield stability [74]. The identification of important genetic markers corresponding to the QTLs co-localized with the target genes provides a basis for the application of this work in plant breeding.

### Conclusion

The methods used in this study could serve as a promising approach for data integration in multiple stress tolerance investigations across species. The information provided adds to the body of knowledge by providing researchers with a unique vanguard integrative data analysis system towards genetic dissection of complex polygenic traits. While the results have shown that genes with functionally relevant across species for multiple stresses have been successfully identified, the study may have more implications in comparative study of major cereal crops, thus providing insight into functional and evolutionary information. These data could, therefore, be used in comparative genomics and in breeding programs towards improving stress tolerance in sorghum and related species.

## Supporting information

S1 Fig Volcano plots for gene expression profiles. This figure shows differential expression of genes with most significant at the top of the plot. The volcano plot represents unpaired t-test based on the evaluation of tissue type contributing to the gene expression (a) and on the evaluation of treatment effect on the experimental samples (b). The red dots indicate a statistical significance for the up and down-regulated genes at the fold-changes, 2, above which all genes have p-value < 0.01 and below which p-value > 0.01. The x-axis represents the log fold change and the y-axis represents the -log10(p-value).
(PDF)

S2 Fig Summarized description of drought related gene-trait associations. This description is based on functional ontology enrichment analysis that include enriched ontology terms from five plant ontologies (GO, TO, EO, GRO and PO). Genes were queried based on their association with the relevant ontology terms in the respective category. Three main GO categories (BP, MF and CC) were used to query the genes corresponding to stress related terms in GO. Only genes supported by all ontology termed from each ontology group were captured. From the pooled total, only unique were selected for gene enrichment analysis based on p-value < 0.01.
(PDF)

S3 Fig **Scatter plot for semantic similarities in enriched GO-terms of the GO main categories biological process (a), cellular component (b) and a summarized description of the GO annotation and classification of the protein sequences based on Blast analysis (c)**. The multidimensional scaling based scatter plot shows semantic similarities in the enriched and non-redundant GO-terms association with a set of drought responsive genes. As multidimensional scaling provides an option of using an eigenvalue of the GO-terms’ pairwise distance matrix, the coordinate position of the GO-terms’ semantic similarities in the enriched genes are displayed to the two-dimensional spaces [41]. The description of the 3 main GO categories (BP, CC and MF) and 28 sub-categories for the GO-terms that were assigned to the sorghum drought responsive genes were shown based on the hits that recovered significant number of genes in the non-redundant database. The x-axis in (c) shows the GO-categories and the y-axis shows the number of genes classified.
(EPS)

S1 Table Description of sorghum drought responsive genes and orthologs conserved in other three species. This supplementary file gives description of sorghum drought responsive genes identified based on drought related GO-terms, sequence similarity search and sorghum orthologous genes in maize, rice and Arabidopsis.
(XLSX)

S2 Table **Description of the gene expression profiles for the up-regulated sorghum drought stress genes for root and shoot tissues (a), for the leaf tissue of 2 genotypes under drought condition (b) and the combined expression profiles of the up-regulated genes for the two data-sets (GSE30249) and (GSE80699) (c)**. The gene expression was determined by Fragments Per Kilobase of transcript per Million mapped reads (RPKM) for the 2 tissues (root and shoot) treated with ABA and for the 2 genotypes (IS20351 and IS22330) leaf tissues treated under drought stress. A detailed description of the gene expression profiles in both data sets provides annotation of the up-down regulated genes based on the non-redundant known database. While the list of expressed genes used in the heat-map and hierarchical clustering with the corresponding GO IDs was based on GSE30249 (a), the list of selected top 500 expressed genes was based on GSE80699 (b). Functional annotation show the GO annotated genes and the corresponding GO IDs for which enriched GO-terms are shown, p-value, FDR < 0.05. The fold change of the up regulated genes of the combined list and the shared (commonly expressed genes) with the corresponding GO annotation and enriched GO-terms are shown, p-value, FDR < 0.05. The sorghum orthologs for the shared genes in other species and their putative function is also indicated.
(XLSX)

S3 Table Maize gene expression data for leaf and ovary tissues based on drought treatment (GSE40070). The table shows a description of the three hundred significantly expressed genes extracted from the total expression for both tissues. Functional gene enrichment and GO annotation (P-value, FDR < 0.01) show enriched genes in the two tissues and a detailed description of gene entries and the corresponding enriched drought associated GO-terms. An expression profile for the most common 22 enriched maize drought associated GO-terms and the corresponding gene and accession IDs are also shown.
(XLSX)

S4 Table **Description of rice gene expression profile for leaf tissue based on drought stress in 3 varieties, GSE57950 (a), for leaf and root tissues based on salinity treatment, GSE73181 (b) and for drought responsive genes that are co-expressed under salt stress (c)**. The genes that were up-regulated under drought condition were evaluated for their responses under salt stress. Significantly expressed genes were used for functional enrichment analysis and GO annotation (p-value, FDR < 0.01). Detail description of enriched gene entries and the corresponding enriched GO-terms based on tissue and treatment groupings are shown for significantly expressed genes under salt condition. An expression profile for the 22 most common enriched rice drought and salt associated GO-terms and the corresponding gene and GO IDs are also provided. Sorghum orthologs and their putative functions are described based on these selected rice genes for which most abundant enriched salt specific GO-terms are given.
(XLSX)

S5 Table Description of sorghum genes responsive to drought and other stresses based on the analysis of GO biological processes. This provides description of the five abiotic stresses including drought, salt, cold, heat and oxidative stress and the associated list of genes expressed. The number and list of common genes across multiple stresses and unique to particular stress are also presented.
(XLSX)

S6 Table Detailed description of sorghum drought responsive genes identified under variable environments as defined by plant ontologies. This description consists of a detailed id list and the corresponding nucleotide sequences of genes identified based on functional ontology enrichment analysis that include enriched ontology terms from five plant ontologies. For further information, refer to the S2 Fig caption.
(XLSX)

S7 Table Protein-sequences used for multiple sequence alignments to generate phylogenetic tree. Four hundred and ninety three protein sequences from the single gene models of the sorghum specific drought responsive genes and orthologs were selected for MSA.
(FASTA)

S8 Table Sorghum orthologous drought responsive genes conserved across species. For further information, refer to the caption given under S6 Table.
(DOC)

S9 Table Sorghum specific drought responsive genes and orthologs conserved in other species. This table shows the description of the total number of conserved orthologs across species out of which a selected number of conserved genes were used for reconstructing phylogenetic tree. The unique conserved genes shown in the list for the purpose of phylogenetic tree corresponds with the gene list whose protein sequences were used for MSA as indicated in S7 Table. List of genes which are exclusively specific to sorghum are also provided.
(XLSX)

S10 Table Nexus formatted phylogenetic tree file with bootstrap values based on 1000 replicates. The description provided presents the gene IDs for the conserved orthologs (single-gene models) and the acronyms used for sorghum orthologs and the bootstrap values for the nodes. The description for the acronyms is provided in S9 Table.
(TXT)

S11 Table Description of the GO functional annotation for the enriched stress responsive genes based on p-value and FDR < 0.05. The gene entries and the corresponding GO-annotation that fall in the yellow highlighted portion returned the enrichment score above p-value, FDR threshold. Some entries that were enriched based on p-value score may have been screened out based on FDR value score.
(XLSX)

S12 Table Background set for the 50 selected enriched GO-terms. (GMT)

S13 Table Background set for the 50 selected enriched genes. (GMT)

S14 Table Fifty selected gene entries and the corresponding enriched GO-terms. (TXT)

S15 Table Sorghum individual level drought responsive genes identified from the whole plant resistance. The description includes the list of genes identified based on drought tolerance, drought avoidance and drought scape with the combined list of non-redundant genes for drought resistance.
(XLSX)

S16 Table Sorghum candidate drought responsive genes associated with drought tolerance QTLs. This table provides the description of selected target sorghum multi-stress tolerance genes which were further identified for co-localization within known QTLs associated with drought tolerance.
(XLSX)

S17 Table Description of the total Blast hits of the currently identified maize drought responsive genes (query) in association with QTLs sequence (subject) based on Gramene database. Selected drought responsive maize target genes were further identified to have association with QTLs controlling different traits. The best Blast hits were selected based on % identity, bitscor, alignment length and e-vale (1e-10) and a single best hit per query gene was selected. The molecular marker IDs and the corresponding QTL IDs are indicated.
(XLSX)

S18 Table Description of the total Blast hits of the currently identified rice salt responsive genes (query) in association with QTLs sequence (subject) based on Gramene database. Selected salt responsive rice target genes were further identified to have association with QTLs controlling different traits and molecular marker linked to the QTLs. The best Blast hits were selected based on % identity, bitscor, alignment length and e-vale (1e-10) and a single best hit per query gene was selected. The molecular marker IDs and the corresponding QTL IDs are shown.
(XLSX)

## Abbreviations

ABA: abscic acid
Blast: Basic Local Alignment Search Tool
BP: biological process
CC: cellular component
DA: Drought Avoidance
DE: Drought Escape
DR: Drought resistance
DT: Drought Tolerance
EO: Environment Ontology
FDR: False Discovery Rate
GEO: Gene Expression Omnibus
GO: Gene Ontology
GRO: Growth Ontology
iTOL: Interactive tree of life
MF: molecular function
NCBI: National Center for Biotechnology Information
PO: Plant Ontology
TO: Trait Ontology
