# Correction: Cross-species multiple environmental stress responses: An integrated approach to identify candidate genes for multiple stress tolerance in sorghum (*Sorghum bicolor* (L.) Moench) and related model species

**DOI:** 10.1371/journal.pone.0197017

**Published:** 2018-05-03

**Authors:** Adugna Abdi Woldesemayat, David M. Modise, Junaid Gamieldien, Bongani K. Ndimba, Alan Christoffels

The third author's name is spelled incorrectly. The correct name is: Junaid Gamieldien. The correct citation is: Woldesemayat AA, Modise DM, Gamieldien J, Ndimba BK, Christoffels A (2018) Cross-species multiple environmental stress responses: An integrated approach to identify candidate genes for multiple stress tolerance in sorghum (*Sorghum bicolor* (L.) Moench) and related model species. PLoS ONE 13(3): e0192678. https://doi.org/10.1371/journal.pone.0192678
