# Supplementary figures and images for: Cross-species multiple environmental stress responses: An integrated approach to identify candidate genes for multiple stress tolerance in sorghum (Sorghum bicolor (L.) Moench) and related model species
Source: PLoS One. 2018 Mar 28;13(3):e0192678. doi: 10.1371/journal.pone.0192678 (PMC5873934; doi:10.1371/journal.pone.0192678)

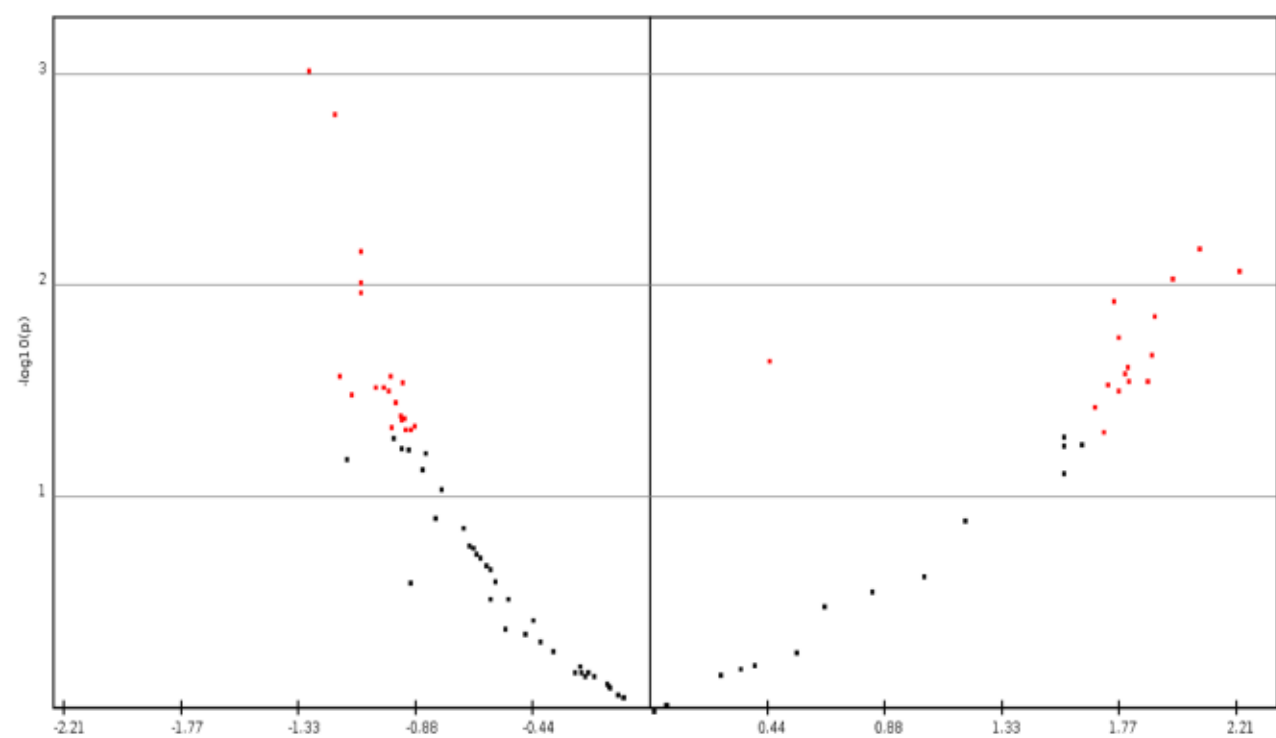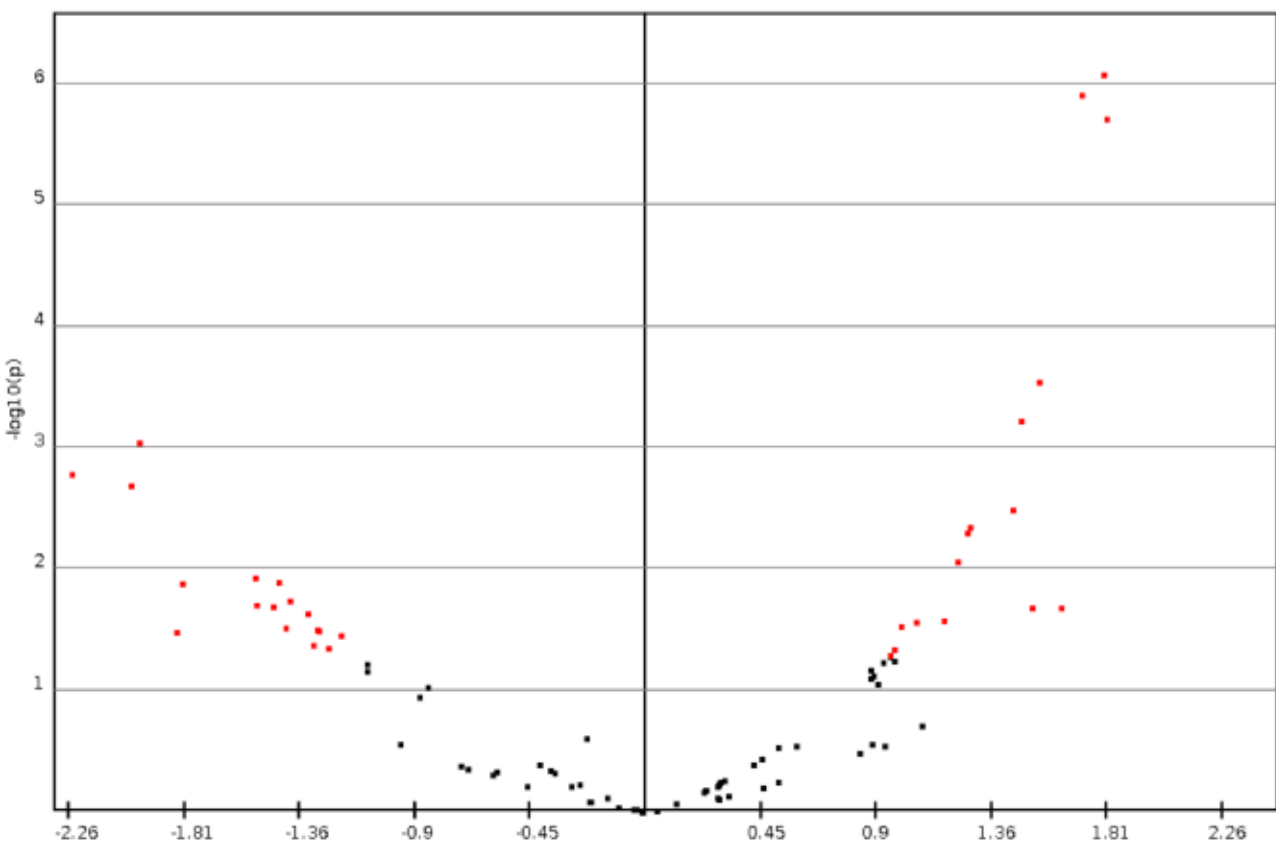

Supplement: S1 Fig — This figure shows differential expression of genes with most significant at the top of the plot. The volcano plot represents unpaired t-test based on the evaluation of tissue type contributing to the gene expression (a) and on the evaluation of treatment effect on the experimental samples (b). The red dots indicate a statistical significance for the up and down-regulated genes at the fold-changes, 2, above which all genes have p-value < 0.01 and below which p-value > 0.01. The x-axis represents the log fold change and the y-axis represents the -log10(p-value). (PDF) [file pone.0192678.s001.pdf]

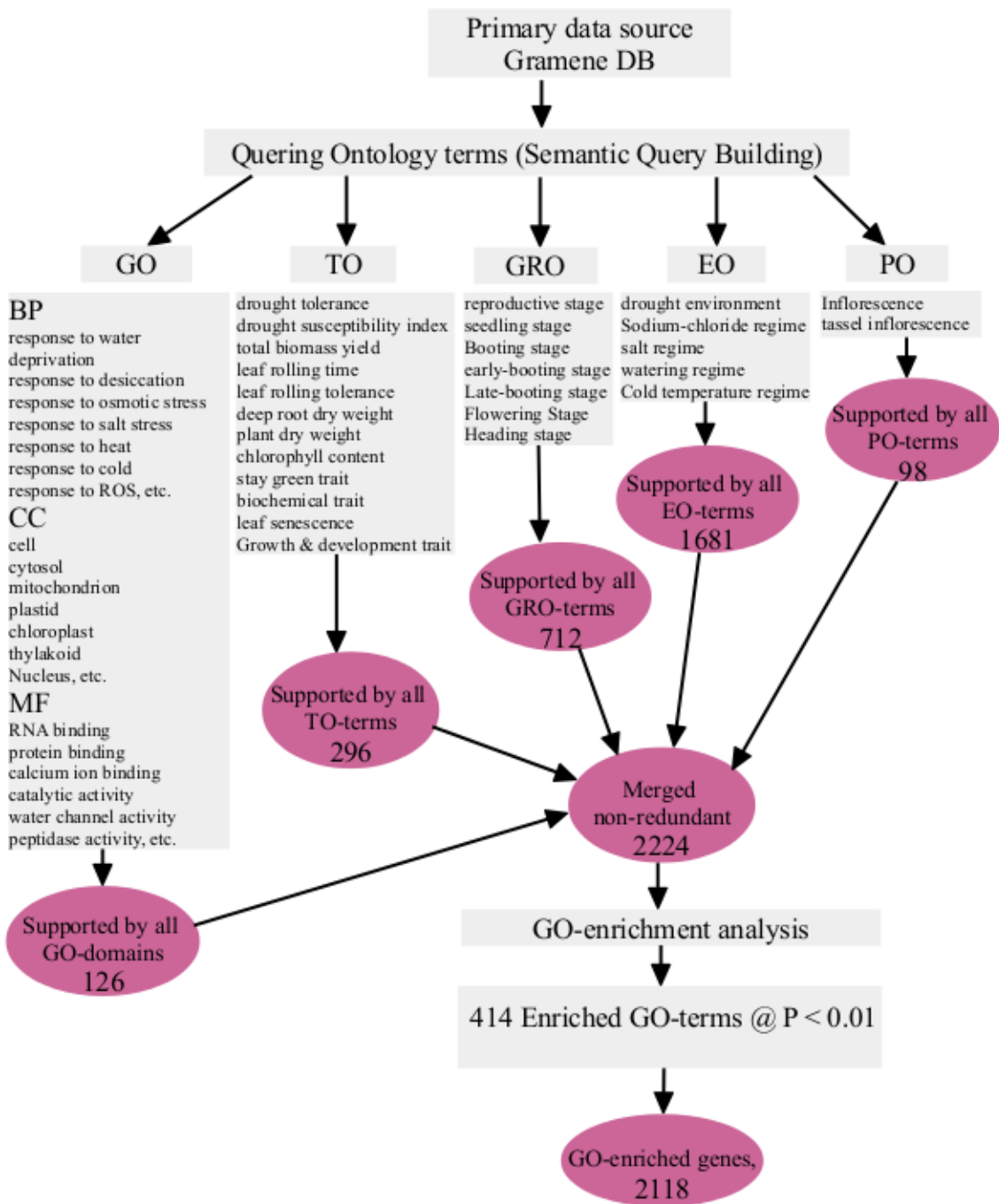

Supplement: S2 Fig — This description is based on functional ontology enrichment analysis that include enriched ontology terms from five plant ontologies (GO, TO, EO, GRO and PO). Genes were queried based on their association with the relevant ontology terms in the respective category. Three main GO categories (BP, MF and CC) were used to query the genes corresponding to stress related terms in GO. Only genes supported by all ontology termed from each ontology group were captured. From the pooled total, only unique were selected for gene enrichment analysis based on p-value < 0.01. (PDF) [file pone.0192678.s002.pdf]
