# Supplementary material for: Cross-species multiple environmental stress responses: An integrated approach to identify candidate genes for multiple stress tolerance in sorghum (Sorghum bicolor (L.) Moench) and related model species
Source: PLoS One. 2018 Mar 28;13(3):e0192678. doi: 10.1371/journal.pone.0192678 (PMC5873934; doi:10.1371/journal.pone.0192678)
Supplement: S8 Table — For further information, refer to the caption given under S6 Table. (DOC) [file pone.0192678.s011.doc]

**S8 Table. Sorghum drought responsive orthologous genes conserved across species**

| **Sorghum orthologs in other species *** | **Initial/combined genes identified **** | **Orthologs conserved**  ******* | **% orthologs**  **conserved** |
| --- | --- | --- | --- |
| SOA | 613 (initial) | 292 | 60.0 |
| SOM | 138 (initial) | 16 | 3.3 |
| SOR | 214 (initial) | 17 | 3.5 |
| Sorghum | 169 (initial) | 9 | 1.8 |
| SOMA | 751 (combined) | 1 | 0.2 |
| SOMR | 352 (combined) | 9 | 1.8 |
| SORA | 827 (combined) | 47 | 9.7 |
| SOMRA | 965 (combined) | 3 | 0.6 |
| Sorghum_SOA | 782 (combined) | 5 | 1.0 |
| Sorghum_SOM | 307 (combined) | 0 | 0.0 |
| Sorghum_SOR | 383 (combined) | 4 | 0.8 |
| Sorghum_SOMA | 920 (combined) | 18 | 3.7 |
| Sorghum_SOMR | 521 (combined) | 13 | 2.7 |
| Sorghum_SORA | 996 (combined) | 16 | 3.3 |
| Sorghum_SOMRA | 1134 (combined) | 37 | 7.6 |

**Key to legend**:

* For the acronyms specified for sorghum orthologs in other species, refer to Fig 6 and S7 Table.

** The initial genes represent drought responsive sorghum orthologs originally identified in the respective species (Fig 6, Sx Table) and the combined genes represent the combination of the initial genes based on the number of species sharing conserved the genes in common.

*** Non-redundant sorghum orthologs conserved in other species.

**** Percent conserved out of the total conserved genes.
